# Surveillance of antibiotic-resistant bacteria integrating whole genome sequencing and epidemiological information – a global, systematic review, 2012–2023

**DOI:** 10.1186/s13756-026-01821-9

**Published:** 2026-09-24

**Authors:** Felix Reichert, Mark Perry, Mirco Sandfort, Sebastian Haller, Dunja Said, Tim Eckmanns, Kevin McDermott, Caro Noake, Robert Wolff, Jörg B. Hans

**Affiliations:** 1 Department of Infectious Disease Epidemiology, Robert Koch Institute, Berlin, Germany; 2 Kleijnen Systematic Reviews, Unit 6, Escrick Business Park, York, YO19 6FD UK

**Keywords:** Whole genome sequencing, WGS, Surveillance, Antimicrobial resistance, Carbapenem, Carbapenemase, Methicillin, Vancomycin, Enterobacterales, Acinetobacter, Staphylococcus aureus, Enterococci

## Abstract

**Background:**

Epidemiologic surveillance is one pillar in the global efforts to stop the spread of antibiotic-resistant bacteria (ARB) and increasingly includes whole genome sequencing (WGS). However, no review systematically examined the characteristics of such surveillance systems until February 2025. We conducted a systematic review to identify and describe routine surveillance systems of ARB, that integrate WGS and epidemiological information.

**Methods:**

A protocol was developed and published on PROSPERO. We searched 8 databases and Google. Included articles needed to report on a national or subnational surveillance system and include epidemiological data as well as WGS of carbapenem-resistant or carbapenemase-producing Enterobacterales (CRE) or Acinetobacter spp., methicillin-resistant Staphylococcus aureus (MRSA) or vancomycin-resistant Enterococci (VRE). Surveillance system characteristics were analysed narratively.

**Results:**

From 13,142 searched articles, 41 were included. The publications were stratified by pathogen. The strata demonstrated similar WGS approaches with a predominance of short-read sequencing platforms. Typing methods mostly included multilocus sequence typing and single nucleotide polymorphism -based analyses for higher discrimination. Reported cluster thresholds showed a wide range of values within and between species. Across strata, there was often a low level of epidemiological data collection, such as exposure of cases, and integration in cluster analyses. WGS surveillance complemented with at least basic epidemiological data has been reported from all six WHO regions for CRE. However, fewer studies and no studies at all for MRSA and VRE were included from the African, Eastern Mediterranean and South-East Asian regions. The national coverage of surveillance ranged from 36% for MRSA to 56% for CRE.

**Conclusion:**

WGS-based surveillance on ARB is employed worldwide with predominant use of short-read technology and largely consistent typing methods. Substantial variation in genomic clustering thresholds underscores the need for internationally harmonized standards for data analysis. Insufficient epidemiological information or lacking integration in the analysis of WGS data may reduce the ability to infer transmission pathways and identify outbreaks. To fully exploit the potential of WGS-based surveillance systems in this context for public health and contain current and prevent future outbreaks, we thus call for a routine collection of detailed epidemiological data.

**Supplementary Information:**

The online version contains supplementary material available at https://doi.org/10.1186/s13756-026-01821-9.

## Introduction

Infections with antibiotic-resistant bacteria (ARB) cause increased morbidity, mortality and healthcare costs. It has been estimated that ARB led to 1.27 million deaths globally in 2019 [1]. Hence, the World Health Organization (WHO) declared antimicrobial resistance, to be “one of the top 10 global public health threats facing humanity” [2]. There are predictions of a ten-fold increase in death due to antibiotic resistant infections by 2050 [3].

In 2015, the WHO launched a global action plan to address antibiotic resistance, aiming to optimize antimicrobial use and strengthen surveillance systems [4]. Surveillance of ARB may be able to detect the emergence and dissemination of new antibiotic-resistant strains. By helping to discern transmission pathways, chains of infection may be stopped more quickly.

Traditional pathogen-based surveillance methods rely on phenotypic antibiotic susceptibility testing (AST) although molecular methods like PCR are also used [5]. Alongside epidemiological information, these methods may support inferences on transmission pathways through the identification of identical resistance patterns and determinants. However, to confirm the genetic relatedness of a suspected outbreak clone, a typing method needs to be deployed. Because of its superior discriminatory power, whole genome sequencing (WGS) has become the gold standard for bacterial strain typing and is increasingly implemented in routine surveillance [6–8].

WGS can provide the complete DNA sequence of a bacterial isolate [9] through the application of different sequencing technologies. In general, available technologies for WGS either generate short (Illumina, Ion Torrent) or long reads (Oxford Nanopore (ONT), Pacific Biosciences). Short-read technologies produce millions of high-quality reads, typically 100–300 base pairs in length, enabling high-throughput sequencing of samples and thus decreasing costs. However, these short reads are generally unable to fully reconstruct genomic structures of interest, including mobile genetic elements such as resistance plasmids. In contrast, long-read technologies can overcome these limitations. Previously, long-read technologies were characterized by higher sequencing error rates as compared to short-read platforms and the limited possibility of multiplexing of samples increased the costs per sequenced isolate [10]. However, recent advancements substantially increased accuracy and affordability of long-read sequencing.

Obtained sequences can be used to reconstruct the genome from scratch (*de novo* assembly) or be aligned against a reference sequence (mapping). Multiple bacterial genomes can be compared to evaluate their genetic relatedness, which is usually measured in the number of differences between isolates. In incrementing order, multilocus sequence typing (MLST) has the lowest discriminatory power because it relies only on the allelic combination of a few house-keeping genes, whose number depends on the species to be analyzed. In contrast, the core genome (cg)MLST is based on the pairwise allelic comparison of thousands of genes present in all isolates of a species and whole genome (wg)MLST which also includes genes of the accessory genome existing only in a subset of isolates. Single nucleotide polymorphisms (SNP)-based analyses provide the highest resolution to differentiate between closely related isolates.

Irrespective of the typing method, the fewer differences, the more recently the strains are likely to have diverged, which in conjunction with epidemiological information may provide important insights into transmission pathways, cluster affiliation of isolates in outbreak settings, or the emergence and dissemination of pathogens [11].

Therefore, the combination of WGS-based pathogen information along with epidemiological data and reasoning is a promising means to make a difference in the prevention of AMR [11, 12]. However, to our knowledge, a comprehensive overview of existing surveillance systems that integrate WGS with epidemiological information is lacking. In order to provide an important resource for health authorities to develop or evaluate their own systems, this systematic review aims to describe the existence, distribution and characteristics of WGS systems, integrated with epidemiological information, used for surveillance of antibiotic-resistant organisms globally.

## Materials and methods

### Protocol

A detailed protocol for this systematic review was developed collaboratively by the systematic-reviewing company (SRC) and the Funding Academic Institution (FAI), and signed off prior to any extraction of data. The protocol was published on (PROSPERO 2023 CRD42023456676) (https://www.crd.york.ac.uk/prospero/display_record.php?ID=CRD42023456676), and is summarised in Table 1 below.

**Table 1:** Systematic review protocol

What are the characteristics of models for routine surveillance of pathogens with antimicrobial resistance, that utilise Whole Genome Sequencing integrated with epidemiological information?	
Population	**Inclusion** • Human samples containing pathogens with antimicrobial resistance. • Only include studies that consider at least one of the following species: • Carbapenem-resistant or carbapenemase-producing Enterobacterales (CRE/CPE) • Carbapenem-resistant or carbapenemase-producing *Acinetobacter* spp. (CRA/CPA) • Methicillin-resistant *Staphylococcus aureus* (MRSA) • Vancomycin-resistant *Enterococci* (VRE) **Exclusion** • Other pathogens / resistances • Studies that ONLY handle food, animal or other environmental samples.
Inclusion criteria relating to content of publications	Any reported models of whole genome sequencing, integrated with epidemiological information, that are used for surveillance of antimicrobial resistance, will be included. The publication must have sufficient detail about methods, to determine that the system described meets the definition of a surveillance system (which will exclude sole outbreak reports). The WHO definition of public health surveillance will be used: ’the continuous, systematic collection, analysis and interpretation of health-related data […] guiding priority-setting and planning and evaluation public health policy and strategies’ [13]. The surveillance system must operate at a regional, national or supra-regional level. There must be detailed description of the population, including epidemiological information (has to include information on patient characteristics, such as age, sex, diagnosis, geographic location, patient location (ward/clinic), specimen type, or clinical outcome). Data covering at least one of the following four pieces of information must be available for inclusion: • Information on any countries that have started to build such systems, or have them already established • Scope of the models: for sporadic outbreak detection, for temporal and/or spatial trends, or routine monitoring of genetic properties • Transmission pathways described. • Visualisation methods applied [trees, timelines, heatmaps, networks and others].
Study types	**Inclusion** • Full-text primary articles of any form (including grey literature) that include a description of WGS models for surveillance of antimicrobial resistance *that are in actual use*. **Exclusion** • Papers solely discussing hypothetical systems that are not currently in use. • Secondary (i.e. review/guideline) articles and abstracts.
Dates	2012 to August 2023 (details see below)
Languages	English, Chinese (Mandarin), French, Spanish and German. Other languages will be excluded.
Stratification	**Unconditional** Stratification will be applied unconditionally^A^ for the type of pathogen (the four categories are given in the population section). **Conditional** Conditional stratification^B^ will occur for the type of additional environmental, or animal samples used (no additional samples versus additional environmental samples versus additional food samples versus additional animal samples versus other categories that reflect combinations of the additional types of sample [for example, additional food and environmental samples]). If no meta-analysis is used, then these conditional categories for additional samples will become unconditional, and the strata of the review will be formed by permutations of pathogen and additional sample categories.
Synthesis of findings	Meta-analysis will be used if appropriate, but it is envisaged that the most likely form of synthesis will be narrative (tabular).

Abbreviations: WHO = World Health Organization

A. unconditional stratification: stratified ‘up front’, prior to any initial overall meta-analysis, and therefore not conditional on any initial overall meta-analysis I^2^ result.

B. conditional stratification: stratified after initial overall meta-analysis shows an I^2^ value of *≥* 50%

### Literature searches

Searches were carried out to identify relevant information on currently used routine surveillance of ARB, that utilise WGS integrated with epidemiological information. The search strategies were developed specifically for each database and the keywords adapted according to their configuration. Searches combined relevant search terms comprising indexed keywords (e.g. Medical Subject Headings (MeSH) and EMTREE) and free text terms appearing in the title and/or abstract of database records. Search terms were identified through discussion with the review team and FAI, by scanning background literature and ‘key articles’ already known to the review team, and by browsing database thesauri.

The search was limited by publication date (2012- August 2023) but not by language, nor publication status.

The following databases were searched for relevant articles:

- MEDLINE and Epub Ahead of Print, In-Process, In-Data-Review & Other Non-Indexed Citations and Daily Update (Ovid): 1946 to 2023/08/21.
- Embase (Ovid): 1974 to 2023/08/21.
- PubMed (https://pubmed.ncbi.nlm.nih.gov/): up to 2023/08/22.
- KSR Evidence (https://ksrevidence.com/): up to 2023/08/30.
- Cochrane Database of Systematic Reviews (CDSR) (Wiley): up to 2023/08/Iss8.
- Cochrane Central Register of Controlled Trials (CENTRAL) (Wiley): up to 2023/07/Iss7.

The following grey literature resources were searched from inception to the present:

- International HTA Database (https://database.inahta.org/): up to 2023/08/29.
- Policy Commons (https://policycommons.net/): up to 2023/08/22.

References in retrieved articles were checked for additional publications. Further to this, a search of the first 100 results retrieved by Google (search terms: ‘whole genome sampling resistance surveillance’ on 08/09/23) was conducted, to ensure that no key grey literature documents were missed.

For all searches undertaken by the SRC Information Team, the main Embase strategy was independently peer-reviewed by a second SRC Information Specialist, based on the Canada’s Drug and Health Technology Agency (CADTH) checklist [14–16].

Details of the search strategies are contained in Additional file 1.

### Methods of article selection, quality assessment, and data extraction

Two SRC reviewers (KMcD and MP) independently selected potentially eligible articles from the titles and abstracts based on the protocol.

Eligibility of the full-texts was assessed by one SRC reviewer (MP) based on the protocol. A second (blinded) one (KMcD), re-evaluated 5% of the full-text papers. The two reviewers obtained 100% concordance. Finally, FAI staff checked full-text paper inclusion and found further relevant articles.

One SRC reviewer (MP) extracted the following data in a tailor-made extraction sheet in Word: location and time of the surveillance, type of surveillance (sentinel or comprehensive), coverage (national or subnational), isolate collection methods, target population, epidemiological data collected (age, sex, diagnosis, outcome, suspected place of acquisition, ward/clinic, travel history, hospitalisation history), and WGS methodologies used. There were no outcome data, as this was a descriptive review. Members of the FAI team checked data extraction accuracy.

No appraisal of bias within studies was carried out, since the surveillance methods and not the study results were described in this systematic review.

### Data synthesis

The articles were stratified by pathogen (carbapenem-resistant or carbapenemase-producing Enterobacterales (CRE/CPE), carbapenem-resistant or carbapenemase-producing *Acinetobacter* spp. (CRA/CPA), methicillin-resistant *Staphylococcus aureus* (MRSA), vancomycin-resistant *Enterococci* (VRE)) as prescribed in the protocol. The extracted characteristics of the underlying surveillance were described and summarised with frequency or range (genetic distance) and median (time) within each stratum. Where adequate, relative frequencies were compared between subgroups (such as WHO region) or summarised across strata. Formal quantitative analysis was not conducted, as this was considered over-analysis and not pre-specified in the protocol. Synthesis was performed by one reviewer (MP), with accuracy checked by the FAI team. The systematic review was written up adhering to PRISMA methodology [17, 18], with the completed PRISMA checklist available in Additional file 2.

## Results

### Included articles

**Fig. 1:**
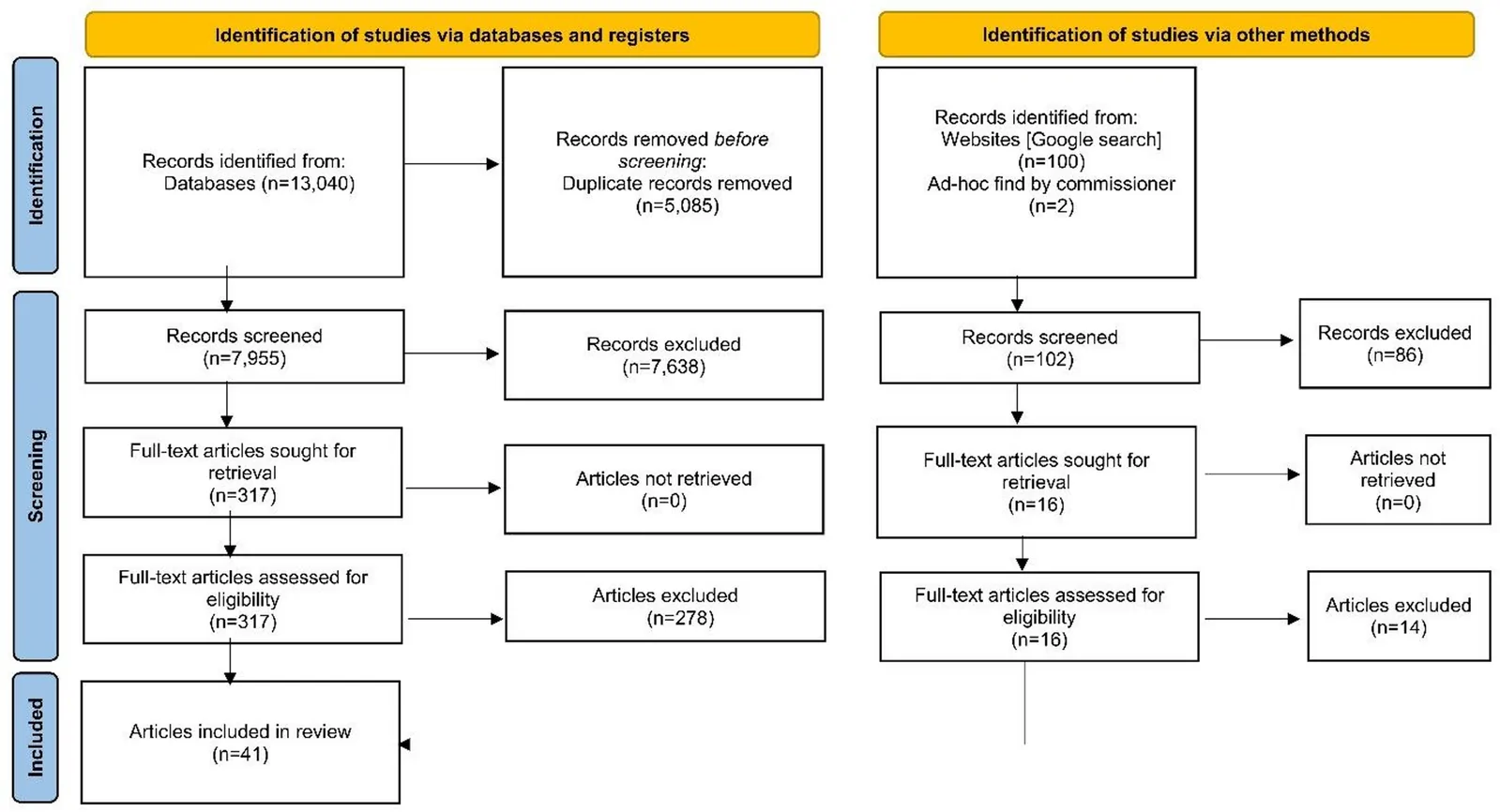
PRISMA flow diagram

The systematic search yielded 13,040 abstracts. After removal of duplicates, 7,955 articles remained. The first 100 ‘titles’ from a Google search were added, alongside two further ad-hoc identified abstracts, making a total of 8,057 abstracts/titles for sifting. From the initial sift, 333 full-text articles were retrieved. Of these, 41 articles [19–59] were included in the review. Exclusion reasons for the 292 articles are provided in Additional file 3. Figure 1 provides the PRISMA flow chart.

### Locations, sectors and target pathogens

**Fig. 2:**
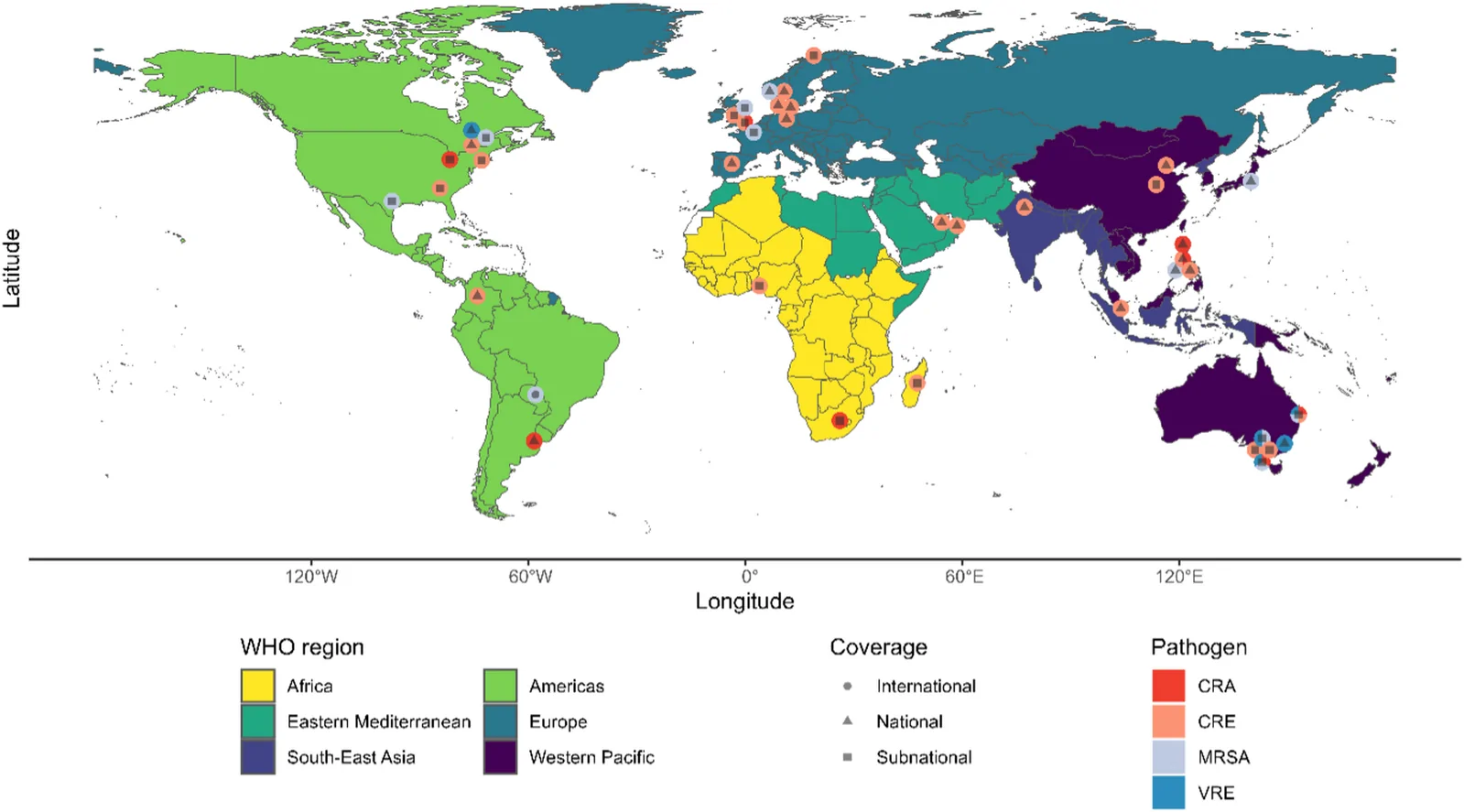
Locations of included articles by coverage, pathogen(s) under surveillance and WHO region

Surveillance including epidemiologic and WGS data was reported from all six WHO regions (Fig. 2). Earliest and 85% of publications originate from the Western Pacific region (*n* = 14), European region (*n* = 11) and region of the Americas (*n* = 10). No article reported additional environmental or animal samples. Table 2 summarises these articles per pathogen stratum. Five articles included pathogens from several strata [22, 30, 52, 53, 56]. Some articles included target pathogens of this systematic review but had a wider or different pathogen scope (*n* = 13; e.g. ESBL-producing Enterobacterales), or focussed on specific properties (*n* = 5; e.g. NDM-5-producing *E. coli*).

### Time and surveillance periods

The first articles were published in 2017 on CRE/CPE and MRSA, and in 2020 for CRA/CPA and VRE with isolates dating back to 2007 and 2013, respectively. Reported surveillance periods varied between 2 and 144 months, with a median of 19 months for MRSA to 36 months for CRE/CPE and VRE (overall 24 months). Surveillance periods of two years or less often resulted from self-contained surveillance or pilot studies [24, 27, 44, 49, 53–56, 58]. Most publications with reporting durations longer than 2 years [19, 20, 23, 25, 29, 30, 32, 33, 35, 36, 38, 39, 41, 43, 47, 48, 51, 52, 59] used datasets from ongoing, continuous surveillance systems.

**Table 2:** Included studies, separated by pathogen stratum

Publication	WHO REGION Country Coverage (type of surveillance) ^$^	Start year End year approx. duration	Target species (sample material)	No. isolates: -study total -belonging to stratum -stratum WGS	Sequencing technology and lab (central, decentral, external)	Typing methods	Availability* of epidemiological information								Epidemiological information integrated with genetic differences (threshold) to infer clusters or transmission pathways	Public availability of sequence data
							Age	Gender	Diagnosis	Outcome	HAI/ CAI	Ward, clinic	Travel	Hosp. history		
Stratum 1: Carbapenem-resistant or carbapenemase-producing *Enterobacterales*																
Afolayan, 2021 [20]	AFRICAN Nigeria Subnational (sentinel, tertiary care hospitals)	2016 2018 36 m	Antimicrobial-resistant *Klebsiella* spp. (clinical: blood, CSF, urine)	141 11 11	Illumina *de novo* central	MLST, SNP	✓	✓	✓	✗	✓	✓	✗	✗	Time of isolate recovery; others for visualisation (ESBL-Kp: 1–3 SNPs)	Yes
Milenkov, 2021 [46]	AFRICAN Madagascar Subnational (sentinel, maternity wards)	2018 2019 9 m	ESBL- producing *Enterobacterales* (screening)	170 1 1	Illumina + ONT *de novo* central	MLST, SNP	✓	✓	✓	✗	✗	✗	✗	✗	Unclear; isolate origin for visualisation (ESBL-Ec: <10 SNPs)	Yes
Karlowsky, 2021 [35]	AMERICAS Canada National (comprehensive, tertiary care hospitals)	2007 2018 144 m	ESBL-positive *Escherichia coli* and *Klebsiella pneumoniae* (clinical)	812 23 23	Illumina *de novo* central	MLST	✓	✓	✗	✗	✗	✓	✗	✗	NR (NA)	Yes
Saavedra, 2021 [51]	AMERICAS Colombia National (comprehensive, NRL)	2013 2017 60 m	Carbapenem-resistant *Klebsiella pneumoniae* (clinical)	425 425 425	Illumina *de novo* central	MLST, SNP	✓	✓	✓	✗	✗	✗	✗	✗	Unclear (NR)	Yes
Bulens, 2023 [23]	AMERICAS USA Subnational (sentinel, MuGSI)	2012 2015 36 m	Carbapenem-resistant *Enterobacterales* (clinical)	1499 1499 12	Illumina *de novo* central	MLST	✓	✓	✓	✓	✓	✗	✓	✓	NR (NA)	No
Lapp, 2023 [39]	AMERICAS USA Subnational (comprehensive, MuGSI and statewide CRE surveillance)	2016 2018 36 m	Carbapenem-resistant (blaKPC-carrying) *Enterobacterales* (clinical)	255 255 255	Illumina *de novo* decentral	MLST, SNP	✓	✓	☑	✗	✗	✓	✗	✓	Comorbidities, geographic (≤ 5, 10, and 15 SNVs)	Yes
Al-Farsi, 2020 [21]	EAST MEDITERRANEAN Oman National (comprehensive)	2015 2015 10 m	*Escherichia coli* with reduced susceptibility to carbapenems (clinical and screening)	35 35 35	Illumina *de novo* central	MLST, cgMLST, wgMLST, SNP	✓	✓	✓	✓	✗	✓	☑	✓	Hospital, ward, isolation date, hospitalisation and travel history (Ec: ≤10 SNPs)	Yes
Sonnevend, 2022 [55]	EAST MEDITERRANEAN United Arab Emirates National (sentinel)	2018 2019 9 m	Carbapenem-resistant *Enterobacterales* (clinical and screening)	504 504 504	Illumina *de novo* central	MLST, cgMLST	✓	✓	✗	✗	✗	✓	✗	✗	Hospital (Kp: ≤15 allelic differences)	Yes
Do Nascimento, 2017 [28]	EUROPE UK Subnational (comprehensive)	2015 2016 18 m	Enteroaggregative *Escherichia coli* (clinical: faecal samples)	155 1 1	Illumina mapping central	MLST	✓	✓	✗	✗	✗	✗	☑	✗	NR (NA)	Yes
Roer, 2017 [50]	EUROPE Denmark National (comprehensive)	2014 2015 24 m	Third-generation cephalosporin-resistant *Escherichia coli* (blood culture)	552 5 5	Illumina *de novo* central	MLST, SNP	✓	✓	✓	✗	✗	✗	✗	✗	Unclear; isolate origin for visualisation (Ec: ≤10 SNPs)	No
Hammerum, 2020 [32]	EUROPE Denmark National (comprehensive)	2016 2019 44 m	OXA-244-producing *Escherichia coli* (clinical and screening)	24 24 24	Illumina *de novo* central	MLST, cgMLST	✓	✓	✗	✗	✗	✓	✓	✓	Travel and hospitalisation history, place of sampling and residence, (Ec: ≤10 allelic differences)	Yes
Taylor, 2021 [56]	EUROPE UK Subnational (sentinel)	2016 2016 6 m	Amikacin-resistant *Enterobacterales* (clinical and screening)	271 135 135	Illumina *de novo* central	MLST	✓	✓	✓	✗	✗	✗	☑	✗	NR (NA)	No
Canada-Garcia, 2022 [24]	EUROPE Spain National (sentinel)	2019 2019 4 m	Carbapenemase-producing *Klebsiella pneumoniae* and *Escherichia coli* (clinical)	403 403 403	Illumina *de novo* central	MLST, SNP, cgMLST	✓	✓	✗	✗	✗	✗	✗	✗	Unclear (Kp: ≤10 SNPs, ≤ 5 allelic differences)	Yes
Hans, 2023 [33]	EUROPE Germany National (comprehensive)	2013 2019 84 m	NDM-5-producing *Escherichia coli* (clinical and screening)	329 329 222	Illumina *de novo* central	MLST, cgMLST, SNP	✓	✓	✗	✗	✗	☑	✗	✗	Geography (Ec: ≤100 SNPs)	Yes
Ljungquist, 2023 [43]	EUROPE Norway National (comprehensive)	2015 2021 84 m	Carbapenemase-producing *Enterobacterales* (clinical and screening)	389 389 389	Illumina *de novo* central	MLST, cgMLST	✓	✓	✗	✗	✗	✗	✓	✗	Hospital, travel history (NR)	Yes
Raffelsberger, 2023 [49]	EUROPE Norway Subnational (comprehensive)	2015 2016 19 m	ESBL-producing *Escherichia coli* and *Klebsiella pneumoniae* (screening)	4999 2 2	Illumina *de novo* external	MLST, SNP	✓	✓	✓	✗	✗	✗	✓	✓	NR (Ec: ≤17 SNPs)	Yes
Nagaraj, 2021 [47]	SOUTH EAST ASIA India National (sentinel)	2013 2019 84 m	Carbapenem resistant *Klebsiella pneumoniae* (clinical)	344 280 280	Illumina *de novo* NR	MLST, SNP	✓	✓	✓	✗	✓	✓	✗	✗	Hospital, inpatient/outpatient, age (Kp: ≤3 SNPs)	Yes
Marimuthu, 2017 [44]	WEST PACIFIC Singapore National (comprehensive)	2013 2015 17 m	Carbapenem-resistant *Enterobacteriaceae* (clinical and screening)	430 430 206	Illumina *de novo* central	MLST, SNP	✓	✓	✓	✓	✗	✓	✓	✓	Hospital (Ec: ≤58 SNPs, Kp: ≤151 SNPs)	Yes
Sherry, 2019 [52]	WEST PACIFIC Australia Subnational (comprehensive)	2012 2016 60 m	Carbapenemase-producing *Enterobacterales* (clinical and screening)	361 361 361	Illumina *de novo* central	MLST, SNP	✓	✓	✗	✗	✓	✓	✓	✓	“Data from concurrent epidemiologic investigations” (Hospital, hospitalisation and travel history) (≤ 23 SNPs)	Yes
Argimon, 2020 [22]	WEST PACIFIC Philippines National (sentinel)	2013 2014 24 m	Carbapenemase- and/or ESBL-producing *Klebsiella pneumoniae* and *Escherichia coli* (clinical)	805 ~ 420 ~ 420	Illumina *de novo* central	MLST, SNP	✓	✗	✗	✗	✓	✓	✗	✗	Hospital, ward isolation date, HAI/CAI, age (threshold unclear)	Yes
Carlos, 2021 [25]	WEST PACIFIC Philippines National (sentinel)	2015 2017 36 m	Carbapenem-, extended spectrum beta-lactam-, and cephalosporin-resistant *Klebsiella pneumoniae* (clinical)	259 139 139	Illumina *de novo* central	MLST, SNP	✓	✓	✗	✗	✓	✓	✗	✗	Hospital, ward, sampling time, age, HAI/CAI (threshold unclear)	Yes
Lane, 2021 [38]	WEST PACIFIC Australia Subnational (comprehensive)	2016 2018 36 m	Carbapenemase-producing *Enterobacterales* (clinical and screening)	402 402 402	Illumina *de novo* central	MLST, SNP	✓	✓	✓	✓	✗	✓	✓	✓	Hospital, hospitalisation and travel history and eventual additional outbreak investigation (threshold unclear)	Yes
Forde, 2023 [30]	WEST PACIFIC Australia Subnational (comprehensive)	2017 2021 50 m	Carbapenemase-producing *Enterobacterales* (clinical and screening)	2660 293 293	Illumina *de novo* central	MLST, SNP	✓	✗	✗	✗	✓	✓	✗	✗	Hospital, HAI/CAI (≤ 5 SNPs/Mb)	Yes
Ko, 2023 [37]	WEST PACIFIC China National (sentinel)	2019 2020 12 m	Carbapenemase-producing *Escherichia coli* (clinical)	31 31 13	ONT *de novo* NR	MLST, cgMLST	✓	✓	✗	✗	✗	✗	✗	✗	Unclear (NR)	No
Li, 2023 [42]	WEST PACIFIC China Subnational (sentinel)	2017 2022 72	Carbapenem-resistant *Klebsiella pneumoniae* (clinical)	662 662 101	Illumina *de novo* external	MLST, SNP	✓	✓	✗	✗	✗	✗	✗	✗	Unclear, age, sex, isolation region and year for visualisation (NR)	Yes
Stratum 2: Carbapenem-resistant or carbapenemase-producing *Acinetobacter* spp.																
Perovic, 2022 [48]	AFRICAN South Africa Subnational (sentinel)	2017 2019 30 m	*Acinetobacter baumannii* complex (focus on colistin resistant) (blood culture)	4822 1786 38	Illumina *de novo* central	MLST, SNP	✓	✓	✓	✓	✗	✓	✗	✓	Hospital and age for visualisation (NR)	Yes
Adams, 2020 [19]	AMERICAS Argentina National (sentinel, NRL)	2014 2017 48 m	NDM-producing *Acinetobacter* spp. (clinical)	40 40 40	Illumina *de novo* NR	MLST, SNP	✓	✓	✗	✗	✗	✓	✗	✗	Hospital but no cluster definition (threshold unclear)	Yes
Iovleva, 2022 [34]	AMERICAS USA Subnational (comprehensive for study network)	2017 2018 14 m	Carbapenem-resistant *Acinetobacter baumannii* (clinical and screening)	155 155 115	Illumina + ONT *de novo* central	MLST, SNP	✓	✓	✓	✓	✗	✓	✗	✗	Hospital, study site (≤ 10 SNPs)	Yes
Taylor, 2021 [56]	EUROPE UK Subnational (sentinel)	2016 2016 6 m	Amikacin- resistant *Acinetobacter baumannii* (clinical and screening)	271 16 16	Illumina *de novo* central	MLST	✓	✓	✓	✗	✗	✗	☑	✗	NR (NA)	No
Argimon, 2020 [22]	WEST PACIFIC Philippines National (sentinel)	2013 2014 24 m	Carbapenemase-producing *Acinetobacter baumannii* (clinical)	805 108 108	Illumina *de novo* central	MLST, SNP	✓	✗	✗	✗	✓	✓	✗	✗	Hospital, ward isolation date, HAI/CAI, age (threshold unclear)	Yes
Chilam, 2021 [26]	WEST PACIFIC Philippines National (sentinel)	2013 2014 24 m	*Acinetobacter baumannii* (focus on carbapenem-resistant) (clinical)	5254 445 117	Illumina *de novo* central	MLST, SNP	✓	✓	✓	✗	✓	✓	✗	✗	Hospital (threshold unclear)	Yes
Sherry, 2021 [53]	WEST PACIFIC Australia Subnational (unclear)	2017 2017 2 m	Carbapenem-resistant *Acinetobacter* spp. (clinical)	408 2 2	Illumina *de novo* central	MLST, SNP	✓	✓	✗	✗	✓	✓	✗	✓	Hospitalisation history, wards, admission dates (≤ 25 SNPs)	Yes
Forde, 2023 [30]	WEST PACIFIC Australia Subnational (comprehensive)	2017 2021 50 m	Carbapenem-resistant *Acinetobacter baumannii* (clinical and screening)	2660 35 35	Illumina *de novo* central	MLST, SNP	✓	✗	✗	✗	✓	✓	✗	✗	Hospital, HAI/CAI (≤ 5 SNPs/Mb)	Yes
Stratum 3: Methicillin-resistant *Staphylococcus aureus*																
Lee, 2017 [40]	AMERICAS USA Subnational (comprehensive, Ten primary care clinics)	2007 2015 108 m	*Staphylococcus aureus* (MRSA and MSSA) (clinical and screening)	144 71 71	Illumina mapping NR	MLST, SNP	✓	✓	✓	✗	✗	✗	✗	✗	Place of sampling (NR)	Yes
Guthrie, 2020 [31]	AMERICAS Canada Subnational (sentinel)	2010 and 2016 24 m	Methicillin-Resistant *Staphylococcus aureus* (clinical)	426 426 426	Illumina mapping central	MLST, SNP	✓	✓	✓	✓	✓	✓	✗	✗	Age, hospital, HAI/CAI (≤ 10 SNPs)	Yes
Di Gregorio, 2023 [27]	AMERICAS South America International (sentinel)	2019 2019 7 m	*Staphylococcus aureus* (MRSA and MSSA) (blood culture)	443 182 165	Illumina *de novo* central	MLST, SNP	✓	✓	✓	✗	✓	✗	✗	✗	Country, HAI/CAI (threshold unclear)	Yes
Toleman, 2019 [57]	EUROPE UK Subnational (comprehensive)	2012 2013 12 m	Methicillin-resistant *Staphylococcus aureus* (MRSA) (blood culture)	425 425 425	Illumina *de novo* central	MLST, SNP	✓	✓	✗	✗	✓	✗	✗	✗	NR	Yes
Enger, 2022 [29]	EUROPE Norway National (comprehensive)	2008 2016 108 m	Methicillin-resistant *Staphylococcus aureus* (clinical and screening)	475 475 108	Illumina *de novo* central	MLST, SNP	✓	✓	✓	✗	✓	✗	☑	✓	Birth country and HAI/CAI for visualisation (NR)	Yes
van der Mee-Marquet, 2022 [58]	EUROPE France Subnational (sentinel)	2021 2021 8 m	*Staphylococcus aureus* (MRSA and MSSA) (blood culture)	53 5 5	Illumina NR	MLST, (SNP unclear)	✓	✓	✓	✓	✗	✓	✗	✗	NR	No
Masim, 2021 [45]	WEST PACIFIC Philippines National (sentinel)	2013 2014 24 m	Methicillin-resistant *Staphylococcus aureus* (clinical)	6211 3549 118	Illumina *de novo* external	MLST, SNP	✓	✓	✗	✗	✓	✓	✗	✗	Unclear (NR)	Yes
Sherry, 2021 [53]	WEST PACIFIC Australia Subnational (unclear)	2017 2017 2 m	Methicillin-resistant *Staphylococcus aureus* (clinical and screening)	408 88 88	Illumina *de novo* central	MLST, SNP	✓	✓	✗	✗	✓	✓	✗	✓	Hospitalisation history, wards, admission dates (≤ 15 SNPs)	Yes
Sherry, 2022 [54]	WEST PACIFIC Australia Subnational (comprehensive)	2017 2018 14 m	Methicillin-resistant *Staphylococcus aureus* (clinical and screening)	2275 811 811	Illumina *de novo* central	MLST, SNP	✓	✓	✗	✗	✓	✓	✗	✓	Hospitalisation history, wards, admission dates (≤ 15 SNPs)	Yes
Yamaguchi, 2022 [59]	WEST PACIFIC Japan National (comprehensive)	2010 2018 56 m**	Methicillin-resistant *Staphylococcus aureus* (clinical: skin and pus)	1770 1770 92	Illumina *de novo* external	MLST, SNP	✓	✓	✗	✗	✗	✗	✗	✗	Unclear (NR)	Yes
Forde, 2023 [30]	WEST PACIFIC Australia Subnational (comprehensive)	2017 2021 50 m	Methicillin-resistant *Staphylococcus aureus* (clinical and screening)	2660 620 620	Illumina *de novo* central	MLST, SNP	✓	✗	✗	✗	✓	✓	✗	✗	Hospital, HAI/CAI (≤ 5 SNPs/Mb)	Yes
Stratum 4: Vancomycin-resistant *Enterococci*																
Kleinman, 2023 [36]	AMERICAS Canada National (sentinel)	2013 2018 72 m	Vancomycin-resistant *Enterococcus* ST1478 (blood culture)	115 115 115	Illumina *de novo* central	MLST, SNP	✓	✓	✓	✓	✓	✓	✗	✓	Patient movement data (hospital, ward) (< 20 SNVs)	No
Lee, 2020 [41]	WEST PACIFIC Australia National (sentinel)	2015 2017 36 m	Vancomycin-resistant *Enterococcus faecium* (blood culture)	1025 537 537	Illumina *de novo* central	MLST, SNP	✓	✓	✗	✓	✗	✗	✗	✗	Unclear (NR)	Yes
Sherry, 2021 [53]	WEST PACIFIC Australia Subnational (unclear)	2017 2017 2 m	*vanA* vancomycin- resistant *Enterococcus faecium* (clinical and screening)	408 64 64	Illumina *de novo* central	MLST, SNP	✓	✓	✗	✗	✓	✓	✗	✓	Hospitalisation history, ward, admission dates (≤ 25 SNPs)	Yes
Sherry, 2022 [54]	WEST PACIFIC Australia Subnational (comprehensive)	2017 2018 14 m	*vanA* vancomycin- resistant *Enterococcus faecium* (clinical and screening)	2275 346 346	Illumina *de novo* central	MLST, SNP	✓	✓	✗	✗	✓	✓	✗	✓	Hospitalisation history, wards, admission dates (≤ 25 SNPs)	Yes
Forde, 2023 [30]	WEST PACIFIC Australia Subnational (comprehensive)	2017 2021 50 m	Vancomycin-resistant *enterococci* (clinical and screening)	2660 433 433	Illumina *de novo* central	MLST, SNP	✓	✗	✗	✗	✓	✓	✗	✗	Hospital, HAI/CAI (≤ 5 SNPs/Mb)	Yes

Abbreviations: bp = base pair; cg = core genome; CSF = cerebrospinal fluid; Ec = Escherichia coli; HAI/ CAI = hospital- /community- acquired infection; Hosp. history = hospitalisation history; Kp = Klebsiella pneumoniae; m = month; med = medium; MLST = multi-locus sequence typing; MuGSI = Multi-site Gram-negative Surveillance Initiative; NR = not reported; PFGE = pulsed field gel electrophoresis; SCCmec = staphylococcal cassette chromosome mec; SNP = single nucleotide polymorphism; target = target pathogen (i.e. MRSA); target WGS = target pathogens submitted for WGS; VNTR = variable number tandem repeat; WGS = whole genome sequencing; WHO = World Health Organization

^$^ “Sentinel” refers to surveillance in a selected sample of sites that do not cover all areas in the national or sub-national region; “comprehensive” refers to surveillance that is potentially including all sites within the covered area

*✓ Available; ☑ Available but reportedly < 80% complete; ✗ Not reported/not available

**Intermittent periods from 2010 to 2018: 2010 (February to September), 2012 (February 2012 to January 2013), 2014 (January to December), 2016

(January to December), and 2018 (January to December)

### Summary of findings

#### Stratum 1: Carbapenem-resistant or carbapenemase-producing Enterobacterales (CRE)

For CRE surveillance, there were 25 publications from 17 countries in all six WHO regions: the African region (Madagascar [46], Nigeria [20]), the region of the Americas (United States of America (USA) [23, 39], Canada [35], Colombia [51]), the South East Asian region (India [47]), the European region (the United Kingdom (UK) [28, 56], Denmark [32, 50], Germany [33], Norway [43, 49], Spain [24]), the Eastern Mediterranean region (United Arabic Emirates (UAE) [55] Oman [21]) and the Western Pacific region (Philippines [22, 25], Australia [30, 38, 52], China [37, 42], and Singapore [44]). National level strategies were reported in 14 publications, operating in Canada [35], China [37], Colombia [51], Denmark [32, 50], Germany [33], India [47], Norway [43], Oman [21], Philippines [22, 25], Singapore [44], Spain [24] and UAE [55]. Eleven of the articles were based on sentinel, rather than comprehensive, surveillance within the subnational or national boundaries (Table 2).

In many cases, collection of data was initiated and coordinated by a national or subnational organisation for AMR surveillance purposes [20–23, 25, 28, 32, 33, 35, 38, 39, 43, 46, 47, 50–52]. These organisations mandated or encouraged participating hospital laboratories to supply relevant isolates, which were then sequenced centrally. In other cases, isolate collection appeared to have an academic or scientific background [24, 30, 37, 42, 44, 49, 55, 56]. Although these two categories did not indicate national or subnational coverage, academic studies tended to include less epidemiological information (63% of academic studies reported ≤ 3 epidemiological variables versus 47% of the reports of ongoing surveillance systems).

Availability and completeness of epidemiological information such as demographic, clinical and exposure data varied widely, with 13/25 studies [22, 24, 28, 30, 33, 35, 37, 42, 43, 46, 50, 51, 55] reporting ≤ 3 epidemiological variables, 7/25 studies [20, 25, 32, 39, 47, 49, 56] reporting 4 to 5, and 5/25 studies [21, 23, 38, 44, 52] reporting ≥ 6 (Table 2).

WGS was conducted for all CRE/CPE isolates in 20 of 25 studies [20–22, 24, 25, 28, 30, 32, 35, 38, 39, 43, 46, 47, 49–52, 55, 56], although this was sometimes unclearly reported. In others, selection criteria were applied, such as additional cephalosporin resistance [23], polymyxin resistance [42], or the availability of geographical information [33]. WGS was typically performed with Illumina technology and *de novo* assembly (22/25) whereas one study used a mapping approach (Table 2). One study conducted hybrid assemblies using Illumina and ONT reads for plasmid reconstruction [46], another ONT reads only for *de novo* assemblies [37] (Table 2). All studies employed MLST genotyping and 21/25 also analyses of higher resolution. SNP-based analyses were the method of choice in 17 studies including three, which also applied cgMLST analyses. Four studies conducted only cgMLST and one compared results from cgMLST, wgMLST and SNP-based analyses [21]. Epidemiological information on exposure (travel, ward or hospitalisation history) was given in 19 studies, enabling transmission analysis of genetic clusters. However, only 14 studies integrated this information for transmission or cluster analyses [20–22, 25, 30, 32, 33, 38, 39, 43, 44, 47, 52, 55]. These were generally based on links in sampling time, ward/hospital location [21, 25] or recent travel [32]). For six and five studies, respectively, this epidemiological information was either not reported or not used for cluster analysis. Genomic cluster thresholds were applied in 14 studies including seven that linked genetic relatedness with epidemiological data. In SNP-based analyses, these cut-offs were either species-specific with values ranging up to 100 SNPs for *E. coli* and up to 151 SNPs for *K. pneumoniae* or were applied irrespective of the species with thresholds of up to 5 SNPs or 5 SNPs/Mb, which considers the size of the reconstructed genomes. In cgMLST analyses, thresholds defining clonally related isolates were 10 and 15 allelic differences for *E. coli* and *K. pneumoniae*, respectively. Overall, 21/25 studies provided sequencing data in publicly available repositories.

#### Stratum 2: Carbapenem-resistant or carbapenemase-producing Acinetobacter spp. (CRA)

Regarding CRA surveillance, 8 publications arose from 6 countries in four WHO regions: the African region (South Africa [48]), the region of the Americas (USA [34], Argentina [19]), the European region (UK [56]) and the Western Pacific region (Philippines [22, 26] and Australia [30, 53]). Both articles from the Philippines [22, 26] and the Argentinian article [19] were at a national level. Within both national and subnational boundaries, 5/8 articles reported sentinel rather than comprehensive surveillance (Table 2).

In 5/8 publications, collection of data was initiated and co-ordinated for AMR surveillance purposes [19, 22, 26, 34, 48]. In other cases, the isolates appeared to be derived from a more academic organisation [30, 53, 56], which was conducting a study outside the remit of an ongoing surveillance system. All three academic studies were subnational, whereas 3/5 of the surveillance system reports were national.

Availability of epidemiological information ranged from 3/8 publications [19, 22, 30] reporting ≤ 3 epidemiological variables, 4/8 publications [26, 34, 53, 56] reporting 4 to 5, and only one article [48] reporting ≥ 6.

Six studies [19, 22, 30, 34, 53, 56] submitted all of their CRA isolates for WGS, whilst two applied additional selection criteria [26, 48]. Sequencing was exclusively conducted with Illumina technology followed by *de novo* assembly including one study, which in combination with ONT reads generated high-quality reference genomes for subsequent SNP calling. Besides MLST employed by all eight studies, SNP-based analyses were performed by seven studies whereas one reported no additional analyses. Transmission or clusters were inferred in 5/8 articles [19, 22, 30, 34, 53] using epidemiological data (such as geography [22]) and genetic information. Among these, three studies applied thresholds (i.e. 10 SNPs, ≤ 25 SNPs or ≤ 5 SNPs/Mb) to identify clonally related isolates. Except for one, all studies provided sequence data in public databases [22].

#### Stratum 3: Methicillin-resistant Staphylococcus aureus (MRSA)

Regarding MRSA surveillance, 11 publications arose from 8 countries and one multi-country collaboration (South America). These were from three WHO regions: the region of the Americas (USA [40], Canada [31] and South America [27]), the European region (UK [57], France [58] and Norway [29]) and the Western Pacific region (Philippines [45], Japan [59] and Australia [30, 53, 54]). National level surveillance was conducted in Norway [29], Japan [59] and the Philippines [45] and involving several countries (international level) in South America [27]. Four publications reported sentinel surveillance (Table 2).

In 5/11 articles [29, 31, 45, 57, 59], data collection was initiated and co-ordinated by an organisation for surveillance purposes. In other cases, the isolates were collected by an academic organisation, operating outside the remit of an ongoing surveillance system [27, 30, 40, 53, 54, 58].

Availability and completeness of epidemiological information varied with 4/11 articles [30, 40, 57, 59] reporting ≤ 3 epidemiological variables, 5/11 studies reporting 4 to 5 [27, 45, 53, 54, 58], and only 2/11 studies [29, 31] reporting ≥ 6.

In 8/11 publications all included MRSA isolates were submitted for WGS. In the others a random subset was selected for WGS [29] or specific criteria applied [45, 59]. WGS always involved short read sequencing using the Illumina technology followed by either *de novo* assembly (eight studies) or mapping (two studies). Predominant typing methods were MLST in combination with SNP-based analyses. Transmission or cluster analyses were reported in 6/11 publications [27, 30, 31, 40, 53, 54], generally based on the interaction of epidemiological factors (such as healthcare setting [31] or geography [40]). In 4/11 publications, cut-off values of ≤ 10 SNPs, ≤ 15 SNPs or ≤ 5 SNPs/Mb defined genomic clusters (Table 2). Generated sequencing data were submitted to public databases by 10/11 studies.

#### Stratum 4: Vancomycin-resistant Enterococci (VRE)

For VRE surveillance, there were 5 publications from two countries and WHO regions: the region of the Americas (Canada [36]), and the Western Pacific region (Australia [30, 41, 53, 54]). National level surveillance was conducted in one of the Australian [41] and the Canadian publication [36] that both collected isolates from sentinel sites. The three other Australian articles rather aimed at a comprehensive subnational coverage [30, 53, 54].

In 2/5 articles [36, 41] data collection was initiated and co-ordinated by a governmental surveillance programme. In the others [30, 53, 54], isolates were collected by an academic organisation.

Availability of epidemiological information varied, with 2/5 articles reporting 3 or fewer epidemiological variables [30, 41], 2/5 articles reporting 4 to 5 [53, 54], and one article reporting 6 or more [36].

In all publications all VRE isolates were submitted for centralised Illumina WGS and *de novo* assembly with subsequent genotyping using MLST and SNP-based analyses. Inferences on transmission or cluster analyses were reported in 4/5 publications [30, 36, 53, 54]. These were generally based on the interaction of epidemiological factors such as location [36], combined with genetic relatedness defined by thresholds of ≤ 20 SNVs, ≤ 25 SNPs or ≤ 5 SNPs/Mb. Sequence data were deposited in public databases by 4/5 articles (Table 2).

## Discussion

Surveillance data including genomic and epidemiological information (integrated genomic surveillance, IGS) was published from all WHO regions. Publications on all four pathogen strata were found from the Region of the Americas and the Western Pacific Region. There were many publications included from the European Region, but none from Eastern Europe. Early and frequent publications originated from countries with high per capita gross domestic product, such as Australia, Singapore, USA, Denmark and UK, or were supported by external sequencing or grants, as was the case in studies from the Philippines. IGS was less often reported in the African, Eastern Mediterranean and South-East Asian Regions. Worldwide coverage varied across pathogen strata. Whilst the 25 reports on CRE crossed all 6 WHO regions, coverage was less global for CRA, MRSA and VRE. In particular, no publication on IGS of VRE outside Australia and Canada met our inclusion criteria, similarly for MRSA across three entire WHO regions. However, we have been made aware of additional surveillance reports in the European Region including IGS on MRSA and VRE that were not identified in the systematic literature search (see limitation section) [60–63]. Given the burden of VRE [64, 65] and MRSA [66, 67] worldwide, widespread surveillance is required. International partners and networks like the Centre for Genomic Pathogen Surveillance or the International Pathogen Surveillance Network [68] can help to establish WGS in LMICs [20, 22]. National WGS data can provide valuable insights but should be integrated with epidemiological data to maximize public health impact and cannot replace AST.

There were further differences between pathogen strata. The proportion of national surveillance ranged from 36% for MRSA to 56% for CRE. Nationwide infection control and antibiotic stewardship interventions are estimated to be more effective than facility-based or regional efforts [69]. There was a similar range of proportions opting for a sentinel approach, from 36% in the MRSA stratum to 63% in the CRA stratum. A sentinel surveillance can provide a nationally representative isolate selection, but was often limited to higher-level healthcare or specific subpopulations.

The reported surveillance does not necessarily reflect the underlying surveillance system. First, only 29/41 publications (71%) describe an underlying surveillance system. Second, surveillance was often enhanced for the reported study period, e.g. by collecting additional case information or conducting WGS on top of traditional pathogen surveillance. Third, WGS was often performed retrospectively so that results could not timely inform the involved stakeholders. For real-time actionability, sequencing needs to be performed with a short sample to feedback-time and case data collection performed routinely.

Regarding epidemiological data, many publications merely included basic demographic information and place of sample collection. Between 36% and 52% of publications in each pathogen stratum only included 3 or less of the 8 epidemiological variables that we extracted. Even if available and reported, exposure information was often not integrated with the genomic data and the integrated cluster or transmission analyses tended to be superficially reported or not reported at all. Integrated cluster analysis was particularly lacking in CRE and MRSA (44 and 45% of articles) and less so in VRE (20% of articles). Nonetheless, some studies applied thresholds for cluster identification and integrated epidemiological information on time (of sampling, etc.), place (ward or hospital, etc.) and person (e.g. age or diagnosis) for transmission analyses. This information is crucial to establish the existence of an outbreak and hint towards the source, e.g. specific procedures in the hospital setting or food items in the outpatient setting. The patient outcome needs to supplement the genetic information when assessing virulence. Reasons for the persistent lack of epidemiologic data integration are not covered in this review but might reflect the lack of beneficial experiences with this relatively new technology and lacking governance for integrated surveillance in the context of a historical coexistence of laboratory-based pathogen surveillance and clinical or public health epidemiology. This could be overcome by collaborative efforts between academic research, hospitals and public health authorities (PHAs) to increase genetic and epidemiologic data completeness and showcase the benefits of integrated analysis. On the technical side, genomic surveillance seems to be often built on existing surveillance systems with separated data storage (siloed IT) and detailed clinical/ epidemiological information often only at hospital level. The enhanced capabilities to detect supraregional outbreaks increase the need to share information between different levels and surveillance systems. Exchange of detailed case information on exposure and outcome from hospitals and timely cluster information will improve integrated outbreak analyses on all levels. In practice, relevant epidemiological data should routinely accompany the isolate or sequence or pre-defined forms or interfaces could be used for enhanced surveillance of cluster isolates. Additionally, academic research can refine integrated analyses as several innovative articles show [30, 34, 38, 39, 52] and close potential gaps in routine surveillance for example regarding pathogens or plasmid outbreaks. Interdisciplinary teams and joint training of public health microbiologists and field epidemiologists [70] as well as providing PHAs with sequences or funding for sequencing could increase data integration. One of the included studies [38] has demonstrated a decrease in KPC-2-producing Enterobacterales infections after a comprehensive prospective genomic and epidemiological surveillance and response program for the control of CPE was released. Additionally, the economic impact of genomic surveillance of ARB on national level should be evaluated further [71, 72].

Integration of animal or environmental samples has not been reported by any included publication. Sequencing of antibiotic-resistant bacteria in animal and food needs to be increased and intersectoral collaboration fostered by political stakeholders [73] in order to identify relevant sources of strain dissemination and outbreaks among humans.

WGS methodology was homogenous across strata, mainly using Illumina short-read technology with a preference for *de novo* assembly. Typing methods mostly included MLST, SNP-based analyses, and cgMLST for CRE. Reported cluster thresholds showed a wide range of values within and between species, reflecting the lack of consensus definitions for clonality between closely related isolates. Across pathogen strata, cut-off values for SNP-based analyses to define genomic clusters ranged ≤ 25 SNPs for CRA, VRE, and MRSA, whereas for CRE a general threshold may not be applicable given the heterogeneity of species. SNP-based analyses typically rely on mapping reads to a given reference. Sequencing errors may appear as false-positive SNPs and mistakenly increase genetic differences. Similarly, the choice of the reference genome profoundly effects the results [74]. A reference sequence phylogenetically too divergent may show genomic characteristics which are not present in the isolates under study. Thus, the absence of SNPs in accessory genomic regions may lead to erroneous homogeneity between isolates, ultimately causing misleading epidemiological inferences. Although beyond the scope of this systematic review, we emphasize the need for international standards, particularly focusing on quality metrics, on how to analyse WGS data, such as percentage of reference sequence covered by isolates in SNP-based analyses. Surveillance systems harmonized in this way worldwide would ensure comparability of data for cross-border evaluation of ARB. Almost all studies in this review shared generated sequence data via public databases which is another important pre-condition for international investigations.

This systematic review is strengthened by the precisely defined and focused protocol. Almost all criteria for inclusion and methodological procedures were decided pre-hoc. The limited initial secondary verification of full-text eligibility assessment and data extraction by the SRC was post-hoc identified as a weakness and therefore supplemented by secondary verification through the FAI. Due to different interpretation of the inclusion criteria ‘detailed description’ of epidemiological data, eight articles [20, 22, 24, 30, 36, 39, 41, 59] were added based on the additional structured full-text screening and suggestion of the FAI after the original paper selection by the SRC, and the initial draft summary. Potential selection bias was minimised by agreement on a common interpretation and final inclusion decision by the SRC.

A risk of bias assessment was deemed irrelevant due to descriptive data. The review did not involve measurement of performance or efficacy and bias can only be manifested by a displacement of a measurement from its ‘true’ state. We were unable to find any useful quality appraisal tools focussing on the reporting quality of papers, even from the Enhancing The Quality and Transparency of Health Research (EQUATOR) network [75], where reporting guidelines were not available for descriptive papers. Formal quality appraisal of the papers was thus omitted. Readers are urged to conduct appropriate checks on relevant studies.

The pre-hoc defined search strategy showed some limitations. After conducting the review, we identified that studies mentioning only carbapenemase-production but not resistance were not found [76, 77]. Also, national surveillance reports were not captured although we searched grey literature sources [60–63]. We anticipated not to include surveillance systems without published results or lacking/incomplete description of the methodology due to the search for published studies and the inclusion criteria. Additionally, inference from the published surveillance results to an underlying surveillance system was not always possible and often challenging. However, the evidence base that has been surveyed is likely to provide an overview of current methodology of IGS systems of ARB with peer-reviewed articles.

For a more comprehensive mapping of all existing IGS systems and their characteristics, we recommend additional screening of the grey literature including national surveillance reports and descriptions of IGS systems, as well as a mapping and direct survey of stakeholders.

## Conclusion

Surveillance of CRE, CRA, MRSA and VRE, integrating WGS with epidemiological information, has been reported globally since 2017 and suggest that WGS is increasingly embedded within operational outbreak detection frameworks. Nevertheless, surveillance appears patchy, with potential for broader national and global coverage, in particular for VRE and MRSA. There is general agreement on technical aspects such as sequencing platforms, *de novo* assembly and sequence typing methods. There is, however, variability in the extent to which epidemiological variables are collected. In many cases, exposure information was not reported or not integrated in the analysis of WGS data. In order to use the potential of WGS to help identify outbreaks and transmission pathways, epidemiological and WGS information have to be adequately integrated into surveillance. This would support Public Health professionals in implementing adequate measures to contain current and prevent future outbreaks.

## Supplementary Information

Below is the link to the electronic supplementary material.

**Supplementary Material 1**: Search strategy.

**Supplementary Material 2**: PRISMA checklist.

**Supplementary Material 3**: List of excluded articles.

## Data Availability

All data generated or analysed during this study are included in this published article [and its additional files].

## Abbreviations

ARB: Antibiotic-resistant bacteria
AST: Antibiotic susceptibility testing
bp: base pair
CADTH: Canada’s Drug and Health Technology Agency
cg: core genome
CPA: Carbapenemase-producing Acinetobacter spp
CPE: Carbapenemase-producing Enterobacterales
CRA: Carbapenem-resistant Acinetobacter spp
CRE: Carbapenem-resistant Enterobacterales
Ec: Escherichia coli
FAI: Funding Academic Institution
HAI/ CAI: Hospital- /community- acquired infection
Kp: Klebsiella pneumoniae
MeSH: Medical Subject Headings
MLST: Multi-locus sequence typing
MRSA: Methicillin-resistant Staphylococcus aureus
MuGSI: Multi-site Gram-negative Surveillance Initiative
NR: Not reported
ONT: Oxford Nanopore Technology
PHA: Public health authority
PFGE: Pulsed field gel electrophoresis
SCCmec: Staphylococcal cassette chromosome mec
SNP: Single nucleotide polymorphism
SRC: Ssystematic-reviewing company
UAE: United Arabic Emirates
UK: United Kingdom
USA: United States of America
VNTR: Variable number tandem repeat
VRE: Vancomycin-resistant Enterococci
wg: whole genome
WGS: Whole genome sequencing
WHO: World Health Organization

## References

[1] Murray CJL, Ikuta KS, Sharara F, Swetschinski L, Robles Aguilar G, Gray A, et al. Global burden of bacterial antimicrobial resistance in 2019: a systematic analysis. Lancet. 2022;399(10325):629–55. 10.1016/S0140-6736(21)02724-0 PMC8841637 35065702

[2] World Health Organization. Antimicrobial resistance (Fact sheet) [Internet] 2021. Available from: https://www.who.int/news-room/fact-sheets/detail/antimicrobial-resistance. Accessed 26 Oct 2023.

[3] The Review on Antimicrobial Resistance. Chaired by Jim O’Neill. Antimicrobial resistance: tackling a crisis for the health and wealth of nations (Attribution 4.0 International (CC BY 4.0)) [Internet]. London: Wellcome Collection; 2014. [accessed 9.4.24].

[4] World Health Organization. Global action plan on antimicrobial resistance [Internet]. Geneva: WHO; 2015. [accessed 11.18.24].

[5] Kaprou GD, Bergspica I, Alexa EA, Alvarez-Ordonez A, Prieto M. Rapid methods for antimicrobial resistance diagnostics. Antibiotics. 2021;10:209. 10.3390/antibiotics10020209 PMC7924329 33672677

[6] Gona F, Comandatore F, Battaglia S, Piazza A, Trovato A, Lorenzin G, et al. Comparison of core-genome MLST, coreSNP and PFGE methods for Klebsiella pneumoniae cluster analysis. Microb Genom. 2020;6(4):000347. 10.1099/mgen.0.000347 PMC7276701 32149598

[7] Price L, Gozdzielewska L, Hendry K, McFarland A, Reilly J. Effectiveness of national and subnational interventions for prevention and control of health-care-associated infections in acute hospitals in high-income and upper-middle-income counties: a systematic review update. Lancet Infect Dis. 2023;23(9):347–60. 10.1016/S1473-3099(23)00049-X 37023784

[8] Price V, Ngwira LG, Lewis JM, Baker KS, Peacock SJ, Jauneikaite E, et al. A systematic review of economic evaluations of whole-genome sequencing for the surveillance of bacterial pathogens. Microb Genom. 2023;9(2):000947. 10.1099/mgen.0.000947 PMC9997737 36790430

[9] World Health Organization. GLASS: whole-genome sequencing for surveillance of antimicrobial resistance. Geneva: WHO; 2020. [accessed 8.9.23].

[10] De Maio N, Shaw LP, Hubbard A, George S, Sanderson ND, Swann J, et al. Comparison of long-read sequencing technologies in the hybrid assembly of complex bacterial genomes. Microb Genom. 2019;5(9). 10.1099/mgen.0.000294 PMC6807382 31483244

[11] Struelens MJ, Sintchenko V, Editorial. Pathogen genomics: empowering infectious disease surveillance and outbreak investigations. Front Public Health. 2020;8:179. 10.3389/fpubh.2020.00179 PMC7248215 32509718

[12] World Health Organization. Global genomic surveillance strategy for pathogens with pandemic and epidemic potential: 2022–2032 [Internet]. Geneva: WHO; 2022. [accessed 31.10.24]. 10.2471/BLT.22.288220 PMC8958828 35386562

[13] World Health Organization. Surveillance in emergencies [Internet]. 2024. Available from: https://www.who.int/emergencies/surveillance. Accessed 28 Oct 2024.

[14] McGowan J, Sampson M, Salzwedel DM, Cogo E, Foerster V, Lefebvre C. PRESS Peer Review of Electronic Search Strategies: 2015 Guideline statement. J Clin Epidemiol. 2016;75:40–6. 10.1016/j.jclinepi.2016.01.021 27005575

[15] Sampson M, McGowan J, Cogo E, Grimshaw J, Moher D, Lefebvre C. An evidence-based practice guideline for the peer review of electronic search strategies. J Clin Epidemiol. 2009;62(9):944–52. 10.1016/j.jclinepi.2008.10.012 19230612

[16] Canadian Agency for Drugs and Technologies in Health. PRESS - Peer Review of Electronic Search Strategies. 2015 Guideline Explanation and Elaboration (PRESS E&E) [Internet]. Ottawa: CADTH; 2016. [accessed 16.11.20].

[17] Page MJ, McKenzie JE, Bossuyt PM, Boutron I, Hoffmann TC, Mulrow CD, et al. The PRISMA 2020 statement: an updated guideline for reporting systematic reviews. BMJ. 2021;372:n71. 10.1136/bmj.n71 PMC8005924 33782057

[18] Page MJ, Moher D, Bossuyt PM, Boutron I, Hoffmann TC, Mulrow CD, et al. PRISMA 2020 explanation and elaboration: updated guidance and exemplars for reporting systematic reviews. BMJ. 2021;372:n160. 10.1136/bmj.n160 PMC8005925 33781993

[19] Adams MD, Pasteran F, Traglia GM, Martinez J, Huang F, Liu C, et al. Distinct mechanisms of dissemination of NDM-1 metallo-beta-lactamase in acinetobacter species in Argentina. Antimicrob Agents Chemother. 2020;64(5):e00324. 10.1128/AAC.00324-20 PMC7179578 32122888

[20] Afolayan AO, Oaikhena AO, Aboderin AO, Olabisi OF, Amupitan AA, Abiri OV, et al. Clones and clusters of antimicrobial-resistant Klebsiella from southwestern Nigeria. Clin Infect Dis. 2021;73(Suppl 4):S308–15. 10.1093/cid/ciab769 PMC8634535 34850837

[21] Al-Farsi HM, Camporeale A, Ininbergs K, Al-Azri S, Al-Muharrmi Z, Al-Jardani A, et al. Clinical and molecular characteristics of carbapenem non-susceptible Escherichia coli: a nationwide survey from Oman. PLoS ONE. 2020;15(10):e0239924. 10.1371/journal.pone.0239924 PMC7546912 33036018

[22] Argimon S, Masim MAL, Gayeta JM, Lagrada ML, Macaranas PKV, Cohen V, et al. Integrating whole-genome sequencing within the national antimicrobial resistance surveillance program in the Philippines. Nat Commun. 2020;11(1):2719. 10.1038/s41467-020-16322-5 PMC7264328 32483195

[23] Bulens SN, Reses HE, Ansari UA, Grass JE, Carmon C, Albrecht V, et al. Carbapenem-resistant enterobacterales in individuals with and without health care risk factors -emerging infections program, United States, 2012–2015. Am J Infect Control. 2023;51(1):70–7. 10.1016/j.ajic.2022.04.003 PMC10881240 35909003

[24] Canada-Garcia JE, Moure Z, Sola-Campoy PJ, Delgado-Valverde M, Cano ME, Gijon D, et al. CARB-ES-19 multicenter study of carbapenemase-producing Klebsiella pneumoniae and Escherichia coli from all Spanish provinces reveals interregional spread of high-risk clones such as ST307/OXA-48 and ST512/KPC-3. Front Microbiol. 2022;13:918362. 10.3389/fmicb.2022.918362 PMC9279682 35847090

[25] Carlos CC, Masim MAL, Lagrada ML, Gayeta JM, Macaranas PKV, Sia SB, et al. Genome sequencing identifies previously unrecognized Klebsiella pneumoniae outbreaks in neonatal intensive care units in the Philippines. Clin Infect Dis. 2021;73(Suppl 4):S316–24. 10.1093/cid/ciab776 PMC8634409 34850834

[26] Chilam J, Argimon S, Limas MT, Masim ML, Gayeta JM, Lagrada ML, et al. Genomic surveillance of Acinetobacter baumannii in the Philippines, 2013–2014. Western Pac Surveill Response J. 2021;12(4):1–15. 10.5365/wpsar.2021.12.4.863 PMC8873916 35251744

[27] Di Gregorio S, Vielma J, Haim MS, Rago L, Campos J, Kekre M, et al. Genomic epidemiology of Staphylococcus aureus isolated from bloodstream infections in South America during 2019 supports regional surveillance. Microb Genom. 2023;9(5):001020. 10.1099/mgen.0.001020 PMC10272885 37227244

[28] Do Nascimento V, Day MR, Doumith M, Hopkins KL, Woodford N, Godbole G, et al. Comparison of phenotypic and WGS-derived antimicrobial resistance profiles of enteroaggregative Escherichia coli isolated from cases of diarrhoeal disease in England, 2015-16. J Antimicrob Chemother. 2017;72(12):3288–97. 10.1093/jac/dkx301 28961934

[29] Enger H, Larssen KW, Damas ES, Aamot HV, Blomfeldt A, Elstrom P, et al. A tale of two STs: molecular and clinical epidemiology of MRSA t304 in Norway 2008–2016. Eur J Clin Microbiol Infect Dis. 2022;41(2):209–18. 10.1007/s10096-021-04353-9 PMC8770451 34687359

[30] Forde BM, Bergh H, Cuddihy T, Hajkowicz K, Hurst T, Playford EG, et al. Clinical implementation of routine whole-genome sequencing for hospital infection control of multi-drug resistant pathogens. Clin Infect Dis. 2023;76(3):e1277–84. 10.1093/cid/ciac726 36056896

[31] Guthrie JL, Teatero S, Hirai S, Fortuna A, Rosen D, Mallo GV, et al. Genomic epidemiology of invasive methicillin-resistant staphylococcus aureus infections among hospitalized individuals in Ontario, Canada. J Infect Dis. 2020;222(12):2071–81. 10.1093/infdis/jiaa147 32432674

[32] Hammerum AM, Porsbo LJ, Hansen F, Roer L, Kaya H, Henius A, et al. Surveillance of OXA-244-producing Escherichia coli and epidemiologic investigation of cases, Denmark, January 2016 to August 2019. Euro Surveill. 2020;25(18):1900742. 10.2807/1560-7917.ES.2020.25.18.1900742 PMC7219033 32400363

[33] Hans JB, Pfennigwerth N, Neumann B, Pfeifer Y, Fischer MA, Eisfeld J, et al. Molecular surveillance reveals the emergence and dissemination of NDM-5-producing Escherichia coli high-risk clones in Germany, 2013 to 2019. Euro Surveill. 2023;28(10):2200509. 10.2807/1560-7917.ES.2023.28.10.2200509 PMC9999457 36892470

[34] Iovleva A, Mustapha MM, Griffith MP, Komarow L, Luterbach C, Evans DR, et al. Carbapenem-resistant Acinetobacter baumannii in U.S. hospitals: diversification of circulating lineages and antimicrobial resistance. mBio. 2022;13(2):e0275921. 10.1128/mbio.02759-21 PMC9040734 35311529

[35] Karlowsky JA, Walkty A, Golden AR, Baxter MR, Denisuik AJ, McCracken M, et al. ESBL-positive Escherichia coli and Klebsiella pneumoniae isolates from across Canada: CANWARD surveillance study, 2007-18. J Antimicrob Chemother. 2021;76(11):2815–24. 10.1093/jac/dkab269 34378029

[36] Kleinman DR, Mitchell R, McCracken M, Hota SS, Golding GR, Smith SW. Vancomycin-resistant Enterococcus sequence type 1478 spread across hospitals participating in the Canadian nosocomial infection surveillance program from 2013 to 2018. Infect Control Hosp Epidemiol. 2023;44(1):17–23. 10.1017/ice.2022.7 35264277

[37] Ko W, Tseng S, Chou C, Li T, Li R, Zhang Y, et al. Molecular epidemiology and comparative genomics of carbapenemase-producing Escherichia coli isolates from 19 tertiary hospitals in China from 2019 to 2020. Front Microbiol. 2023;14:1056399. 10.3389/fmicb.2023.1056399 PMC10160391 37152734

[38] Lane CR, Brett J, Schultz M, Gorrie CL, Stevens K, Cameron DRM, et al. Search and contain: impact of an integrated genomic and epidemiological surveillance and response program for control of carbapenemase-producing enterobacterales. Clin Infect Dis. 2021;73(11):e3912–20. 10.1093/cid/ciaa972 PMC8662772 32663248

[39] Lapp Z, Octaria R, O’Malley SM, Nguyen TN, Wolford H, Crawford R, et al. Distinct origins and transmission pathways of blaKPC enterobacterales across three U.S. states. J Clin Microbiol. 2023;61(8):e0025923. 10.1128/jcm.00259-23 PMC10446861 37439675

[40] Lee GC, Dallas SD, Wang Y, Olsen RJ, Lawson KA, Wilson J, et al. Emerging multidrug resistance in community-associated Staphylococcus aureus involved in skin and soft tissue infections and nasal colonization. J Antimicrob Chemother. 2017;72(9):2461–8. 10.1093/jac/dkx200 PMC5890715 28859442

[41] Lee T, Pang S, Stegger M, Sahibzada S, Abraham S, Daley D, et al. A three-year whole genome sequencing perspective of Enterococcus faecium sepsis in Australia. PLoS ONE. 2020;15(2):e0228781. 10.1371/journal.pone.0228781 PMC7021281 32059020

[42] Li Z, Liu X, Lei Z, Li C, Zhang F, Wu Y, et al. Genetic diversity of polymyxin-resistance mechanisms in clinical isolates of carbapenem-resistant Klebsiella pneumoniae: a multicenter study in China. Microbiol Spectr. 2023;11(2):e0523122. 10.1128/spectrum.05231-22 PMC10100843 36847569

[43] Ljungquist O, Haldorsen B, Pontinen AK, Janice J, Josefsen EH, Elstrom P, et al. Nationwide, population-based observational study of the molecular epidemiology and temporal trend of carbapenemase-producing Enterobacterales in Norway, 2015 to 2021. Euro Surveill. 2023;28(27):2200774. 10.2807/1560-7917.ES.2023.28.27.2200774 PMC10370044 37410381

[44] Marimuthu K, Venkatachalam I, Khong WX, Koh TH, Cherng BPZ, Van La M, et al. Clinical and molecular epidemiology of carbapenem-resistant enterobacteriaceae among adult inpatients in Singapore. Clin Infect Dis. 2017;64(Suppl 2):S68–75. 10.1093/cid/cix113 28475792

[45] Masim ML, Argimon S, Espiritu HO, Magbanua MA, Lagrada ML, Olorosa AM, et al. Genomic surveillance of methicillin-resistant Staphylococcus aureus in the Philippines, 2013–2014. Western Pac Surveill Response J. 2021;12(1):6–16. 10.5365/wpsar.2020.11.1.004 PMC8143927 34094618

[46] Milenkov M, Rasoanandrasana S, Rahajamanana LV, Rakotomalala RS, Razafindrakoto CA, Rafalimanana C, et al. Prevalence, risk factors, and genetic characterization of extended-spectrum beta-lactamase Escherichia coli isolated from healthy pregnant women in Madagascar. Front Microbiol. 2021;12:786146. 10.3389/fmicb.2021.786146 PMC8740230 35003019

[47] Nagaraj G, Shamanna V, Govindan V, Rose S, Sravani D, Akshata KP, et al. High-resolution genomic profiling of carbapenem-resistant Klebsiella pneumoniae isolates: a multicentric retrospective Indian study. Clin Infect Dis. 2021;73(Suppl 4):S300–7. 10.1093/cid/ciab767 PMC8634558 34850832

[48] Perovic O, Duse A, Chibabhai V, Black M, Said M, Prentice E, et al. Acinetobacter baumannii complex, national laboratory-based surveillance in South Africa, 2017 to 2019. PLoS ONE. 2022;17(8):e0271355. 10.1371/journal.pone.0271355 PMC9352035 35926057

[49] Raffelsberger N, Buczek DJ, Svendsen K, Smabrekke L, Pontinen AK, Lohr IH, et al. Community carriage of ESBL-producing Escherichia coli and Klebsiella pneumoniae: a cross-sectional study of risk factors and comparative genomics of carriage and clinical isolates. mSphere. 2023;8(4):e0002523. 10.1128/msphere.00025-23 PMC10470604 37306968

[50] Roer L, Hansen F, Thomsen MCF, Knudsen JD, Hansen DS, Wang M, et al. WGS-based surveillance of third-generation cephalosporin-resistant Escherichia coli from bloodstream infections in Denmark. J Antimicrob Chemother. 2017;72(7):1922–9. 10.1093/jac/dkx092 28369408

[51] Saavedra SY, Bernal JF, Montilla-Escudero E, Arevalo SA, Prada DA, Valencia MF, et al. Complexity of genomic epidemiology of carbapenem-resistant Klebsiella pneumoniae isolates in Colombia urges the reinforcement of whole genome sequencing-based surveillance programs. Clin Infect Dis. 2021;73(Suppl 4):S290–9. 10.1093/cid/ciab777 PMC8634422 34850835

[52] Sherry NL, Lane CR, Kwong JC, Schultz M, Sait M, Stevens K, et al. Genomics for molecular epidemiology and detecting transmission of carbapenemase-producing enterobacterales in Victoria, Australia, 2012 to 2016. J Clin Microbiol. 2019;57(9). 10.1128/JCM.00573-19 PMC6711911 31315956

[53] Sherry NL, Lee RS, Gorrie CL, Kwong JC, Stuart RL, Korman TM, et al. Pilot study of a combined genomic and epidemiologic surveillance program for hospital-acquired multidrug-resistant pathogens across multiple hospital networks in Australia. Infect Control Hosp Epidemiol. 2021;42(5):573–81. 10.1017/ice.2020.1253 34008484

[54] Sherry NL, Gorrie CL, Kwong JC, Higgs C, Stuart RL, Marshall C, et al. Multi-site implementation of whole genome sequencing for hospital infection control: a prospective genomic epidemiological analysis. Lancet Reg Health West Pac. 2022;23:100446. 10.1016/j.lanwpc.2022.100446 PMC9019234 35465046

[55] Sonnevend A, Abdulrazzaq N, Ghazawi A, Thomsen J, Bharathan G, Makszin L, et al. The first nationwide surveillance of carbapenem-resistant Enterobacterales in the United Arab Emirates - increased association of Klebsiella pneumoniae CC14 clone with Emirati patients. Int J Infect Dis. 2022;120:103–12. 10.1016/j.ijid.2022.04.034 35470020

[56] Taylor E, Bal AM, Balakrishnan I, Brown NM, Burns P, Clark M, et al. A prospective surveillance study to determine the prevalence of 16S rRNA methyltransferase-producing Gram-negative bacteria in the UK. J Antimicrob Chemother. 2021;76(9):2428–36. 10.1093/jac/dkab186 34142130

[57] Toleman MS, Reuter S, Jamrozy D, Wilson HJ, Blane B, Harrison EM, et al. Prospective genomic surveillance of methicillin-resistant Staphylococcus aureus (MRSA) associated with bloodstream infection, England, 1 October 2012 to 30 September 2013. Euro Surveill. 2019;24(4):1800215. 10.2807/1560-7917.ES.2019.24.4.1800215 PMC6351993 30696529

[58] n der Mee-Marquet N, Dos Santos S, Diene SM, Duflot I, Mereghetti L, Valentin AS, et al. Strong biofilm formation and low cloxacillin susceptibility in biofilm-growing CC398 Staphylococcus aureus responsible for bacteremia in French intensive care units, 2021. Microorganisms. 2022;10(9):1857. 10.3390/microorganisms10091857 PMC9504214 36144459

[59] Yamaguchi T, Nakamura I, Sato T, Ono D, Sato A, Sonoda S, et al. Changes in the genotypic characteristics of community-acquired methicillin-resistant Staphylococcus aureus collected in 244 medical facilities in Japan between 2010 and 2018: a nationwide surveillance. Microbiol Spectr. 2022;10(4):e0227221. 10.1128/spectrum.02272-21 PMC9431082 35758725

[60] The Danish Integrated Antimicrobial Resistance Monitoring and Research Programme (DANMAP). Reports [Internet]. 2025. Available from: https://www.danmap.org/reports. Accessed 23 Mar 2025. 9725793

[61] Swedres-Svarm. Sales of antibiotics and occurrence of antibiotic resistance in Sweden [Internet]. Solna/Uppsala: Swedres-Svarm; 2023. [accessed 25.3.25].

[62] NORM/NORM-VET. Usage of antimicrobial agents and occurrence of antimicrobial resistance in Norway [Internet]. Tromsø/Ås/Oslo: NORM/NORM-VET; 2023. [accessed 25.3.25].

[63] Stichting Werkgroep Antibioticabeleid (SWAB). NethMap 2024: Consumption of antimicrobial agents and antimicrobial resistance among medically important bacteria in the Netherlands in 2023 [Internet]. SWAB; 2024. Accessed 25 Mar 2025.

[64] Alemayehu T, Hailemariam M. Prevalence of vancomycin-resistant Enterococcus in Africa in one health approach: a systematic review and meta-analysis. Sci Rep. 2020;10(1):20542. 10.1038/s41598-020-77696-6 PMC7688635 33239734

[65] Eichel VM, Last K, Brühwasser C, von Baum H, Dettenkofer M, Götting T, et al. Epidemiology and outcomes of vancomycin-resistant enterococcus infections: a systematic review and meta-analysis. J Hosp Infect. 2023;141:119–28. 10.1016/j.jhin.2023.09.008 37734679

[66] Global burden of bacterial antimicrobial resistance. in 2019: a systematic analysis. Lancet (London England). 2022;399(10325):629–55. 10.1016/S0140-6736(21)02724-0 PMC8841637 35065702

[67] Hasanpour AH, Sepidarkish M, Mollalo A, Ardekani A, Almukhtar M, Mechaal A, et al. The global prevalence of methicillin-resistant Staphylococcus aureus colonization in residents of elderly care centers: a systematic review and meta-analysis. Antimicrob Resist Infect Control. 2023;12(1):4. 10.1186/s13756-023-01210-6 PMC9884412 36709300

[68] World Health Organization. International Pathogen Surveillance Network (IPSN) [Internet] [accessed 26.3.25] [Available from: https://www.who.int/initiatives/international-pathogen-surveillance-network

[69] Slayton RB, Toth D, Lee BY, Tanner W, Bartsch SM, Khader K, et al. Vital Signs: estimated effects of a coordinated approach for action to reduce antibiotic-resistant infections in health care facilities - United States. MMWR Morb Mortal Wkly Rep. 2015;64(30):826–31. PMC4654955 26247436

[70] European centre for disease prevention and control. Fellowship programme: EPIET/EUPHEM [Internet]. 2025. Available from: https://www.ecdc.europa.eu/en/epiet-euphem. Accessed 25 Mar 2025.

[71] Tran M, Smurthwaite KS, Nghiem S, Cribb DM, Zahedi A, Ferdinand AD, et al. Economic evaluations of whole-genome sequencing for pathogen identification in public health surveillance and health-care-associated infections: a systematic review. Lancet Microbe. 2023;4(11):e953–62. 10.1016/S2666-5247(23)00180-5 37683688

[72] Price V, Ngwira LG, Lewis JM, Baker KS, Peacock SJ, Jauneikaite E, et al. A systematic review of economic evaluations of whole-genome sequencing for the surveillance of bacterial pathogens. Microb Genom. 2023;9(2). 10.1099/mgen.0.000947 PMC9997737 36790430

[73] World Health Organization. Tackling antimicrobial resistance (AMR) together. Working Paper 1.0: Multisectoral coordination [Internet]. Geneva: WHO; 2018. accessed 26.3.25.

[74] Olson ND, Lund SP, Colman RE, Foster JT, Sahl JW, Schupp JM, et al. Best practices for evaluating single nucleotide variant calling methods for microbial genomics. Front Genet. 2015;6:235. 10.3389/fgene.2015.00235 PMC4493402 26217378

[75] Equator Network. Enhancing the QUAlity and Transparency Of health Research [Internet] Oxford: Centre for Statistics in Medicine (CSM), NDORMS, University of Oxford. Available from: https://www.equator-network.org/. Accessed 30 Oct 2023.

[76] Hansen F, Porsbo LJ, Frandsen TH, Kaygisiz ANS, Roer L, Henius AE, et al. Characterisation of carbapenemase-producing Acinetobacter baumannii isolates from Danish patients 2014–2021: detection of a new international clone - IC11. Int J Antimicrob Agents. 2023;62(2):106866. 10.1016/j.ijantimicag.2023.106866 37244424

[77] Hammerum AM, Lauridsen CAS, Blem SL, Roer L, Hansen F, Henius AE, et al. Investigation of possible clonal transmission of carbapenemase-producing Klebsiella pneumoniae complex member isolates in Denmark using core genome MLST and National Patient Registry Data. Int J Antimicrob Agents. 2020;55(5):105931. 10.1016/j.ijantimicag.2020.105931 32135203

