# Supplementary material for: Surveillance of antibiotic-resistant bacteria integrating whole genome sequencing and epidemiological information – a global, systematic review, 2012–2023
Source: Antimicrob Resist Infect Control. 2026 Sep 24;15:120. doi: 10.1186/s13756-026-01821-9 (PMC13615607; doi:10.1186/s13756-026-01821-9)
Supplement: Supplementary file 1 [file 13756_2026_1821_MOESM1_ESM.pdf]

Additional file 1 for the article:

"Surveillance of antibiotic-resistant bacteria integrating whole genome sequencing and epidemiological information – a global, systematic review, 2012-2023."

SEARCH STRATEGIES

The search was developed in response to the protocol for this systematic review, but in addition it was designed to meet the demands of a different protocol for a separate but related review. Although the related review is beyond the scope of this report, it should be noted that aspects of the search strategy were influenced by the need to simultaneously meet the needs of both protocols.

Embase (Ovid): 1974 to 2023/08/21
Searched 22.8.23

Antimicrobial resistance + WGS + surveillance systems (no date limit)
OR
Named pathogens + antimicrobial resistance surveillance (2012-C)

1 *Antibiotic sensitivity/ or *Antibiotic resistance/ (88890)
2 ((Antimicrob$ or "anti microbial" or "anti microbials") adj3 (resist$ or susceptib$ or sensitiv$)).ti,ab,ot. (70373)
3 ((Antibiotic$ or "anti biotic" or "anti biotics") adj3 (resist$ or susceptib$ or sensitiv$)).ti,ab,ot. (110423)
4 (AMR or AMU).ti,ab,ot. (14004)
5 exp *Antibiotic Agent/ or *Antiinfective agent/ (696892)
6 (resist$ or susceptib$ or sensitiv$).ti,ab,ot. (3836046)
7 5 and 6 (188044)
8 1 or 2 or 3 or 4 or 7 (319651)
9 exp *whole genome sequencing/ (8405)
10 (genom$ adj3 sequenc$).ti,ab,ot. (148915)
11 DNA sequencing.ti,ab,ot. (40401)
12 WGS.ti,ab,ot. (10912)
13 (genomic adj2 (approach$ or perspective$ or surveill$)).ti,ab,ot. (6884)
14 *gene sequence/ (20912)
15 *gene identification/ or *genetic identification/ or *genome analysis/ (26783)
16 or/9-15 (228347)
17 exp *epidemiological surveillance/ (53104)
18 (surveill$ or monitor$ or predict$ or prevent$).ti,ab,ot. (6181628)
19 17 or 18 (6208786)
20 8 and 16 and 19 (3783)
21 (*Enterococci* or Enterococcus or enterobacter$ or *Klebsiella* or Escherichia or Citrobacter or Serratia or *Acinetobacter*$ or Staphylococc$ or salmonella or shigella).ti,ab,ot. (647632)
22 exp **Enterobacterales/* or exp **Acinetobacter/* or exp **Staphylococcus aureus/* or exp *Staphylococcus/ (287528)
23 21 or 22 (705871)
24 ((antimicrob$ resist$ or anti-microb$ resist$ or antibiotic$ resist$ or anti-biotic$ resist$) adj4 (surveill$ or monitor$ or predict$ or prevent$)).ti,ab,ot. (6106)
25 23 and 24 (2935)
26 limit 25 to yr="2012 -Current" (2186)
27 20 or 26 (5657)

MEDLINE(R) and Epub Ahead of Print, In-Process, In-Data-Review & Other Non-Indexed Citations and Daily (Ovid): 1946 to 2023/08/21
Searched 22.8.23

1 exp drug resistance, microbial/ (183433)
2 ((Antimicrob$ or "anti microbial" or "anti microbials") adj3 (resist$ or susceptib$ or sensitiv$)).ti,ab,ot. (57431)
3 ((Antibiotic$ or "anti biotic" or "anti biotics") adj3 (resist$ or susceptib$ or sensitiv$)).ti,ab,ot. (90177)
4 (AMR or AMU).ti,ab,ot. (9065)
5 exp Anti-Bacterial Agents/ (818432)
6 (resist$ or susceptib$ or sensitiv$).ti,ab,ot. (3092869)
7 5 and 6 (223374)
8 1 or 2 or 3 or 4 or 7 (359756)
9 exp Whole Genome Sequencing/ (17252)
10 (genom$ adj3 sequenc$).ti,ab,ot. (136765)
11 DNA sequencing.ti,ab,ot. (31562)
12 WGS.ti,ab,ot. (7262)
13 (genomic adj2 (approach$ or perspective$ or surveill$)).ti,ab,ot. (5761)
14 or/9-13 (180754)
15 exp Population Surveillance/ (74535)
16 (surveill$ or monitor$ or predict$ or prevent$).ti,ab,ot. (4665141)
17 15 or 16 (4698505)
18 8 and 14 and 17 (3983)
19 exp Enterobacteriaceae/ or exp *Acinetobacter*/ or exp Staphylococcus/ or exp Enterococcus/ (557290)
20 (*Enterococci* or Enterococcus or enterobacter$ or *Klebsiella* or Escherichia or Citrobacter or Serratia or *Acinetobacter*$ or Staphylococc$ or salmonella or shigella).ti,ab,ot. (595407)
21 19 or 20 (769135)
22 ((antimicrob$ resist$ or anti-microb$ resist$ or antibiotic$ resist$ or anti-biotic$ resist$) adj4 (surveill$ or monitor$ or predict$ or prevent$)).ti,ab,ot. (4772)
23 21 and 22 (2322)
24 limit 23 to yr="2012 -Current" (1759)
25 18 or 24 (5461)

Cochrane Central Register of Controlled Trials (CENTRAL) (Wiley): up to 2023/07/Iss7
Cochrane Database of Systematic Reviews (CDSR) (Wiley): up to 2023/08/Iss8
Searched 22.8.23

#1 MeSH descriptor: [Drug Resistance, Microbial] explode all trees 2793
#2 ((Antimicrob* or "anti microbial" or "anti microbials") near/3 (resist* or susceptib* or sensitiv*)):ti,ab 1242
#3 ((Antibiotic* or "anti biotic" or "anti biotics") near/3 (resist* or susceptib* or sensitiv*)):ti,ab 2685
#4 (AMR or AMU):ti,ab 383
#5 MeSH descriptor: [Anti-Bacterial Agents] explode all trees 15226
#6 (resist* or susceptib* or sensitiv*):ti,ab 156699
#7 #5 and #6 3689
#8 #1 or #2 or #3 or #4 or #7 8199
#9 MeSH descriptor: [Whole Genome Sequencing] explode all trees 106
#10 (genom* near/3 sequenc*):ti,ab 669
#11 (DNA sequencing):ti,ab 1822
#12 WGS:ti,ab 140

#13 (genomic near/2 (approach* or perspective* or surveill*)):ti,ab 27
#14 #9 or #10 or #11 or #12 or #13 2439
#15 MeSH descriptor: [Population Surveillance] explode all trees 779
#16 (surveill* or monitor* or predict* or prevent*):ti,ab 374444
#17 #15 or #16 374809
#18 #8 and #14 and #17 36
#19 MeSH descriptor: [Enterobacteriaceae] explode all trees 1839
#20 MeSH descriptor: [*Acinetobacter*] explode all trees 84
#21 MeSH descriptor: [Staphylococcus] explode all trees 1539
#22 MeSH descriptor: [Enterococcus] explode all trees 427
#23 (*Enterococci* or Enterococcus or enterobacter* or *Klebsiella* or Escherichia or Citrobacter or Serratia or *Acinetobacter** or Staphylococc* or salmonella or shigella):ti,ab 8809
#24 #19 or #20 or #21 or #22 or #23 9824
#25 ((antimicrob* resist* or anti-microb* resist* or antibiotic* resist* or anti-biotic* resist*) near/4 (surveill* or monitor* or predict* or prevent*)):ti,ab 3317
#26 #24 and #25 244
#27 #18 or #26 275

CDSR=8
CENTRAL=267

KSR Evidence (https://ksrevidence.com/): up to 2023/08/30 Searched 30.8.23

1 (Antimicrob* or "anti microbial" or "anti microbials") adj3 (resist* or susceptib* or sensitiv*) in All text 759 results
2 (Antibiotic or "anti biotic" or "anti biotics") adj3 (resist* or susceptib* or sensitiv*) in All text 826 results
3 AMR or AMU in Title or Abstract 175 results
4 #1 or #2 or #3 in All text 1359 results
5 (surveill* or monitor* or predict* or prevent*) in All text 62317 results
6 #5 and #4 in All text 532 results
7 genome or genomic or gene or genes or WGS or DNA in Title or Abstract 5653 results
8 #7 and #4 in All text 111 results
9 #8 or #6 in All text 597 results
10 (*Enterococci* or Enterococcus or enterobacter* or *Klebsiella* or Escherichia or Citrobacter or Serratia or *Acinetobacter** or Staphylococc* or salmonella or shigella) in All text 1547 results
11 ("antimicrob* resist*" or "anti-microb* resist*" or "antibiotic* resist*" or "anti-biotic* resist*") adj4 (surveill* or monitor* or predict* or prevent*) in All text 66 results
12 #10 and #11 in All text 27 results
13 #9 or #12 in All text 597 results

PubMed (https://pubmed.ncbi.nlm.nih.gov/): up to 2023/08/22 Searched 22.3.23

#20 Search: #18 and #19 874
#19 Search: (((pubstatusaheadofprint[sb] OR publisher[sb] OR pubmednotmedline[sb]))) 5,407,881
#18 Search: #8 and #14 and #17 3,934
#17 Search: #15 or #16 4,747,906
#16 Search: surveill*[Title/Abstract] OR monitor*[Title/Abstract] OR predict*[Title/Abstract] OR prevent*[Title/Abstract] 4,714,840
#15 Search: "Population Surveillance"[Mesh] 74,523
#14 Search: #9 or #10 or #11 or #12 or #13 150,746

#13 Search: "genome sequenc*"[Title/Abstract] OR "genomic sequenc*"[Title/Abstract] 104,744
#12 Search: "genomic approach*"[Title/Abstract] OR "genomic perspective*"[Title/Abstract] OR "genomic surveil*"[Title/Abstract] 4,746
#11 Search: WGS[Title/Abstract] 7,860
#10 Search: "DNA sequencing"[Title/Abstract] 32,503
#9 Search: "Whole Genome Sequencing"[Mesh] 17,232
#8 Search: #1 or #2 or #3 or #4 or #7 341,688
#7 Search: #5 and #6 199,487
#6 Search: (resist* or susceptib* or sensitiv*) 3,481,326
#5 Search: "Anti-Bacterial Agents"[Mesh] 448,178
#4 Search: (AMR[Title/Abstract] OR AMU[Title/Abstract]) 9,622
#3 Search: "Antibiotic resist*"[Title/Abstract] OR "Anti-biotic resist*"[Title/Abstract] OR "Antibiotic susceptib*"[Title/Abstract] OR "Anti-biotic susceptib*"[Title/Abstract] OR "Antibiotic sensitiv*"[Title/Abstract] OR "Anti-biotic sensitiv*"[Title/Abstract] 74,912
#2 Search: "Antimicrobial resist*"[Title/Abstract] OR "Anti microbial resist*"[Title/Abstract] OR "Antimicrobial susceptib*"[Title/Abstract] OR "Anti microbial susceptib*"[Title/Abstract] OR "Antimicrobial sensitiv*"[Title/Abstract] OR "Anti microbial sensitiv*"[Title/Abstract] 53,983
#1 Search: "Drug Resistance, Microbial"[Mesh] 183,403

INAHTA (https://database.inahta.org/): up to 2023/08/29
Searched 29.8.23

(((Population Surveillance)[mh] OR (surveill* OR monitor* OR predict* OR prevent*)[Title] OR (surveill* OR monitor* OR predict* OR prevent*)[abs]) AND ((genomic* OR gene* OR genome OR WGS OR DNA)[Title] OR (genomic* OR gene* OR genome OR WGS OR DNA)[abs] OR (Whole Genome Sequencing)[mh]) AND ((Drug Resistance, Microbial)[mh] OR (antimicrob* resist* OR anti-microb* resist* OR antibiotic* resist* OR anti-biotic* resist*)[Title] OR (antimicrob* resist* OR anti-microb* resist* OR antibiotic* resist* OR anti-biotic* resist*)[abs]))

Policy Commons (https://policycommons.net/): up to 2023/08/22
Searched 22.8.23

Limits Publication type: Report

Search for title:("antimicrobial resistance" OR "anti-microbial resistance" OR "antimicrobial resistant" OR "anti-microbial resistant" OR "antibiotic resistance" OR "anti-biotic resistance" OR "antibiotic resistant" OR "anti-biotic resistant") AND title:(surveil OR surveillance OR monitor OR monitoring OR prediction OR predict OR prevent OR prevention OR genome OR genomic OR gene OR genes OR WGS)

117 results
