# Supplementary material for: Surveillance of antibiotic-resistant bacteria integrating whole genome sequencing and epidemiological information – a global, systematic review, 2012–2023
Source: Antimicrob Resist Infect Control. 2026 Sep 24;15:120. doi: 10.1186/s13756-026-01821-9 (PMC13615607; doi:10.1186/s13756-026-01821-9)
Supplement: Supplementary file 3 [file 13756_2026_1821_MOESM3_ESM.pdf]

Additional file 3 for the article:

"Surveillance of antibiotic-resistant bacteria integrating whole genome sequencing and epidemiological information – a global, systematic review, 2012-2023."

Exclusion list

Full reference	Reason for exclusion
Aanensen DM, Feil EJ, Holden MTG, Dordel J, Yeats CA, Fedosejev A, et al. Whole-genome sequencing for routine pathogen surveillance in public health: A population snapshot of invasive *Staphylococcus aureus* in Europe. mBio 2016; 7(3):e00444-16	Inadequate population details
Aanensen DM, Carlos CC, Donado-Godoy P, Okeke IN, Ravikumar KL. Implementing Whole-Genome Sequencing for Ongoing Surveillance of Antimicrobial Resistance: Exemplifying Insights Into *Klebsiella pneumoniae*. Clin Infect Dis 2021; 73(Suppl_4):S255-S257	Inadequate population details
Abe R, Akeda Y, Sakamoto N, Kumwenda G, Sugawara Y, Yamamoto N, et al. Genomic characterisation of a novel plasmid carrying blaIMP-6 of carbapenem-resistant *Klebsiella pneumoniae* isolated in Osaka, Japan. Journal of Global Antimicrobial Resistance 2020; 21:195-199	Inadequate population details
Abe R, Akeda Y, Sakamoto N, Takeuchi D, Sugawara Y, Yamamoto N, et al. A Nationwide Plasmidome Surveillance in Thailand Reveals a Limited Variety of New Delhi Metallo-beta-Lactamase-Producing Carbapenem-Resistant Enterobacteriaceae Clones and Spreading Plasmids. J Clin Microbiol 2022; 60(12):e0108022	Inadequate population details
Abe R, Akeda Y, Takeuchi D, Sakamoto N, Sugawara Y, Yamamoto N, et al. Clonal dissemination of carbapenem-resistant *Klebsiella pneumoniae* ST16 co-producing NDM-1 and OXA-232 in Thailand. JAC Antimicrobial Resistance 2022; 4(4):dlac084	Inadequate population details
Abe R, Akeda Y, Sakamoto N, Takeuchi D, Sugawara Y, Yamamoto N, et al. A Nationwide Plasmidome Surveillance in Thailand Reveals a Limited Variety of New Delhi Metallo-beta-Lactamase-Producing Carbapenem-Resistant Enterobacteriaceae Clones and Spreading Plasmids. J Clin Microbiol 2022; 60(12):e0108022	Duplicate of #1207
Afolayan AO, Aboderin AO, Oaikhena AO, Odih EE, Ogunleye VO, Adeyemo AT, et al. An ST131 clade and a phylogroup A clade bearing an O101-like O-antigen cluster predominate among bloodstream *Escherichia coli* isolates from South-West Nigeria hospitals. Microbial Genomics 2022; 8(12):000863	Three hospitals only; population details sparse and not available for all those with isolates
Ahmed MO, Elramalli AK, Baptiste KE, Daw MA, Zorgani A, Brouwer E, et al. Whole Genome Sequence Analysis of the First Vancomycin-Resistant Enterococcus faecium Isolates from a Libyan Hospital in Tripoli. Microbial Drug Resistance-Mechanisms Epidemiology & Disease 2020; 26(11):1390-1398	Single hospital

**Full reference**	**Reason for exclusion**
Al Fadhli AH, Mouftah SF, Jamal WY, Rotimi VO, Ghazawi A. Cracking the Code: Unveiling the Diversity of Carbapenem-Resistant *Klebsiella pneumoniae* Clones in the Arabian Peninsula through Genomic Surveillance. Antibiotics 2023; 12:1081	Inadequate population details
Almeida SCG, Lo SW, Hawkins PA, Gladstone RA, Cassiolato AP, Klugman KP, et al. Genomic surveillance of invasive streptococcus *pneumoniae* isolates in the period pre-pcv10 and post-pcv10 introduction in brazil. Microbial Genomics 2021; 7(10):000635	Streptococcus *pneumoniae*
Al-Mogbel MS, Al-Rashid F, Meqdam M, Al-Ajlan H, Khan MA. Molecular Characterization of *Staphylococcus aureus* Isolated from Renal Hemodialysis (HD) Patients from Saudi Arabia. Journal of Pure and Applied Microbiology 2019; 13(1):265-269	Not a surveillance study
Annavajhala MK, Kelly NE, Geng W, Ferguson SA, Giddins MJ, Grohs EC, et al. Genomic and Epidemiological Features of Two Dominant Methicillin-Susceptible *Staphylococcus aureus* Clones from a Neonatal Intensive Care Unit Surveillance Effort. mSphere 2022; 7(6):e0040922	Inadequate population details
Antonelli A, Giani T, Coppi M, Di Pilato V, Arena F, Colavecchio OL, et al. *Staphylococcus aureus* from hospital-acquired pneumonia from an Italian nationwide survey: activity of ceftobiprole and other anti-staphylococcal agents, and molecular epidemiology of methicillin-resistant isolates. J Antimicrob Chemother 2019; 74(12):3453-3461	Inadequate population details
Arca-Suarez J, Rodino-Janeiro BK, Perez A, Guijarro-Sanchez P, Vazquez-Ucha JC, Cruz F, et al. Emergence of 16S rRNA methyltransferases among carbapenemase-producing *Enterobacterales* in Spain studied by whole-genome sequencing. Int J Antimicrob Agents 2022; 59(1):106456	Not available at British Library (only held to 2016)
Arena F, Menchinelli G, Di Pilato V, Torelli R, Antonelli A, Henrici De Angelis L, et al. Resistance and virulence features of hypermucoviscous *Klebsiella pneumoniae* from bloodstream infections: Results of a nationwide Italian surveillance study. Front Microbiol 2022; 13:983294	No details of population characteristics
Argimon S, David S, Underwood A, Abrudan M, Wheeler NE, Kekre M, et al. Rapid Genomic Characterization and Global Surveillance of *Klebsiella* Using Pathogenwatch. Clin Infect Dis 2021; 73(Suppl_4):S325-S335	Inadequate population details
Asbell PA, DeCory HH. Antibiotic resistance among bacterial conjunctival pathogens collected in the Antibiotic Resistance Monitoring in Ocular Microorganisms (ARMOR) surveillance study. PLoS One 2018; 13(10):e0205814	Insufficient population information for isolates with relevant pathogens
Ashton PM, Owen SV, Kaindama L, Rowe WPM, Lane CR, Larkin L, et al. Public health surveillance in the UK revolutionises our understanding of the invasive Salmonella Typhimurium epidemic in Africa. Genome Med 2017; 9(1):92	Inadequate population details
Auguet OT, Niehus R, Gweon HS, Berkley JA, Waichungo J, Njim T, et al. Population-level faecal metagenomic profiling as a tool to predict antimicrobial resistance in *Enterobacterales* isolates causing invasive infections: An exploratory study across Cambodia, Kenya, and the U*K.* EClinicalMedicine 2021; 36:100910	Inadequate population details
Babiker A, Bower CW, Satola SW, Jacob JT, Woodworth MH. Clinical and Genomic Epidemiology of mcr-9 Containing Carbapenemresistant *Enterobacterales* Isolates in Metropolitan Atlanta, 2012-2017. Open Forum Infect Dis 2021; 8(SUPPL 1):S490	Conference poster

**Full reference**	**Reason for exclusion**
Balushi MA, Kumar R, Al-Rashdi A, Ratna A, Al-Jabri A, Al-Shekaili N, et al. Genomic analysis of the emerging carbapenem-resistant *Klebsiella pneumoniae* sequence type 11 harbouring *Klebsiella pneumoniae* carbapenemase (KPC) in Oman. Journal of Infection & Public Health 2022; 15(10):1089-1096	Outbreak report at single hospital
Becker L, Kaase M, Pfeifer Y, Fuchs S, Reuss A, von Laer A, et al. Genome-based analysis of Carbapenemase-producing *Klebsiella pneumoniae* isolates from German hospital patients, 2008-2014. Antimicrob 2018; 7:62	Inadequate population details
Bibbolino G, Di Lella FM, Oliva A, Lichtner M, Del Borgo C, Raponi G, et al. Molecular epidemiology of NDM-5-producing *Escherichia coli* high-risk clones identified in two Italian hospitals in 2017-2019. Diagn Microbiol Infect Dis 2021; 100(4):115399	Two hospitals only - not regional
Birgy A, Madhi F, Jung C, Levy C, Cointe A, Bidet P, et al. Diversity and trends in population structure of ESBL-producing Enterobacteriaceae in febrile urinary tract infections in children in France from 2014 to 2017. J Antimicrob Chemother 2020; 75(1):96-105	Inadequate population details
Bogaerts B, Nouws S, Verhaegen B, Denayer S, Van Braekel J, Winand R, et al. Validation strategy of a bioinformatics whole genome sequencing workflow for Shiga toxin-producing *Escherichia coli* using a reference collection extensively characterized with conventional methods. Microbial Genomics 2021; 7(3):000531	Inadequate population details
Bordier M, Goutard FL, Antoine-Moussiaux N, Pham-Duc P, Lailler R, Binot A. Engaging Stakeholders in the Design of One Health Surveillance Systems: A Participatory Approach. Frontiers in Veterinary Science 2021; 8:646458	Commentary on engaging stakeholders
Bosse JT, Li Y, Rogers J, Fernandez Crespo R, Li Y, Chaudhuri RR, et al. Whole genome sequencing for surveillance of antimicrobial resistance in actinobacillus pleuro*pneumoniae*. Front Microbiol 2017; 8:311	Animal isolates of non-protocol pathogen
Boula A, Senghore M, Ngoh R, Tassadjo F, Fonkoua MC, Nzouankeu A, et al. Hospital-based Surveillance Provides Insights Into the Etiology of Pediatric Bacterial Meningitis in Yaounde, Cameroon, in the Post-Vaccine Era. Clin Infect Dis 2019; 69(Suppl 2):S148-S155	Non-protocol pathogens
Bovo F, Amadesi S, Palombo M, Lazzarotto T, Ambretti S, Gaibani P. Clonal dissemination of *Klebsiella pneumoniae* resistant to cefiderocol, ceftazidime/avibactam, meropenem/vaborbactam and imipenem/relebactam co-producing KPC and OXA-181 carbapenemase. JAC Antimicrobial Resistance 2023; 5(4):dlad099	Inadequate population details
Brehony C, Lanigan D, Carroll A, McNamara E. Establishment of sentinel surveillance of human clinical campylobacteriosis in Ireland. Zoonoses Public Health 2021; 68(2):121-130	Inadequate population details
Calero-Caceres W, Ortuno-Gutierrez N, Sunyoto T, Gomes-Dias CA, Bastidas-Caldes C, Ramirez MS, et al. Whole-genome sequencing for surveillance of antimicrobial resistance in Ecuador: present and future implications. Rev Panam Salud Publica 2023; 47:e8	Commentary
Calix JJ, de Almeida MSC, Potter RF, Wallace MA, Burnham CAD, Dantas G. Outpatient clonal propagation propelled rapid regional establishment of an emergent carbapenem-resistant *Acinetobacter baumannii* lineage ST499Pas. medRxiv 2022;	No details of population (only gender)
Calix JJ, de Almeida MCS, Potter RF, Wallace MA, Burnham CD, Dantas G. Outpatient Clonal Propagation and Rapid Regional Establishment of an Emergent Carbapenem-Resistant *Acinetobacter* baumannnii Lineage Sequence Type 499Pas. J Infect Dis 2023; 227(5):631-640	No details of population (only gender)

**Full reference**	**Reason for exclusion**
Canada-Garcia JE, Grippo N, de Arellano ER, Bautista V, Lara N, Navarro AM, et al. Phenotypic and molecular characterization of IMP-producing *Enterobacterales* in Spain: Predominance of IMP-8 in *Klebsiella pneumoniae* and IMP-22 in Enterobacter roggenkampii. Front Microbiol 2022; 13:1000787	Inadequate population details
Canton R, Loza E, Aznar J, Castillo FJ, Cercenado E, Fraile-Ribot PA, et al. Monitoring the antimicrobial susceptibility of Gram-negative organisms involved in intraabdominal and urinary tract infections recovered during the SMART study (Spain, 2016 and 2017). Rev Esp Quimioter 2019; 32(2):145-155	Inadequate population details
Canton R, Loza E, Arcay RM, Cercenado E, Castillo FJ, Cisterna R, et al. Antimicrobial activity of ceftolozane-tazobactam against *Enterobacterales* and Pseudomonas aeruginosa recovered during the Study for Monitoring Antimicrobial Resistance Trends (SMART) program in Spain (2016-2018). Rev Esp Quimioter 2021; 34(3):228-237	Inadequate population details
Carter C, Hutchison A, Rudder S, Trotter E, Waters EV, Elumogo N, et al. Uropathogenic *Escherichia coli* population structure and antimicrobial susceptibility in Norfolk, U*K.* J Antimicrob Chemother 2023; 78(8):2028-2036	Inadequate population details
Castanheira M, Deshpande LM, Doyle TB, Mendes RE, Sader HS. Occurrence of beta-Lactamases among *Enterobacterales* Isolated from United States Hospitals: Results of the INFORM Surveillance Program for Ceftazidime-Avibactam. Open Forum Infect Dis 2021; 8(SUPPL 1):S730-S731	Conference abstract
Castanheira M, Kantro V, Hubler C, Mendes RE, Shortridge D. Frequency of Carbapenemases in United States Hospitals (2016-2020) and Activity of Meropenem-Vaborbactam and Comparator Agents Tested Against These Isolates. Open Forum Infect Dis 2022; 9(Supplement 2):S112	Conference abstract
Cave R, Ter-Stepanyan MM, Kotsinyan N, Mkrtchyan HV. An Emerging Lineage of Uropathogenic Extended Spectrum beta-Lactamase *Escherichia coli* ST127. Microbiol 2022; 10(6):e0251122	Inadequate population details
Ceron S, Salem-Bango Z, Contreras DA, Ranson EL, Yang S. Clinical and Genomic Characterization of Carbapenem-Resistant *Klebsiella pneumoniae* with Concurrent Production of NDM and OXA-48-like Carbapenemases in Southern California, 2016-2022. Microorganisms 2023; 11(7):1717	Not a surveillance study
Chan KG. Whole-genome sequencing in the prediction of antimicrobial resistance. Expert Rev Anti Infect Ther 2016; 14(7):617-9	Editorial/commentary/narrative review
Chan WS, Au CH, Ho DN, Chan TL, Ma ES, Tang BS. Prospective study on human fecal carriage of Enterobacteriaceae possessing mcr-1 and mcr-2 genes in a regional hospital in Hong Kong. BMC Infect Dis 2018; 18(1):81	Insufficient population details (only age)
Chang SM, Chen JW, Tsai CS, Ko WC, Scaria J, Wang JL. Antimicrobial-Resistant *Escherichia coli* Distribution and Whole-Genome Analysis of Sequence Type 131 *Escherichia coli* Isolates in Public Restrooms in Taiwan. Front Microbiol 2022; 13:864209	Insufficient population details (only gender)
Charalampous T, Alcolea-Medina A, Snell LB, Williams TGS, Batra R, Alder C, et al. Evaluating the potential for respiratory metagenomics to improve treatment of secondary infection and detection of nosocomial transmission on expanded COVID-19 intensive care units. Genome Med 2021; 13(1):182	Not a surveillance study

**Full reference**	**Reason for exclusion**
Chen FJ, Huang WC, Liao YC, Wang HY, Lai JF, Kuo SC, et al. Molecular Epidemiology of Emerging Carbapenem Resistance in *Acinetobacter* nosocomialis and *Acinetobacter* pittii in Taiwan, 2010 to 2014. Antimicrob Agents Chemother 2019; 63(4):e02007	Insufficient population details
Chen B, Berglund B, Wang S, Borjesson S, Bi Z, Nilsson M, et al. Rapid increase in occurrence of carbapenem-resistant Enterobacteriaceae in healthy rural residents in Shandong Province, China, from 2015 to 2017. Journal of Global Antimicrobial Resistance 2022; 28:38-42	Inadequate population details
Cheng Q, Ma Z, Gong Z, Liang Y, Guo J, Ye X, et al. Whole-genome sequencing analysis of *Klebsiella aerogenes* among men who have sex with men in Guangzhou, China. Front Microbiol 2023; 14:1102907	Inadequate population details
Chilam J, Argimon S, Limas MT, Masim ML, Gayeta JM, Lagrada ML, et al. Genomic surveillance of Pseudomonas aeruginosa in the Philippines, 2013-2014. Western pac 2021; 12(2):4-18	Non-protocol pathogen
Cohen LM, Bosse JT, Stegger M, Li Y, Langford PR, Kielland C, et al. Comparative Genome Sequence Analysis of Actinobacillus pleuro*pneumoniae* Serovar 8 Isolates From Norway, Denmark, and the United Kingdom Indicates Distinct Phylogenetic Lineages and Differences in Distribution of Antimicrobial Resistance Genes. Front Microbiol 2021; 12:729637	Animal isolates only
Collineau L, Bourely C, Rousset L, Berger-Carbonne A, Ploy MC, Pulcini C, et al. Towards One Health surveillance of antibiotic resistance: characterisation and mapping of existing programmes in humans, animals, food and the environment in France, 2021. Euro Surveill 2023; 28(22)	Inadequate population details
Coolen JPM, Jamin C, Savelkoul PHM, Rossen JWA, Wertheim HFL, Matamoros SP, et al. Centre-specific bacterial pathogen typing affects infection-control decision making. Microbial Genomics 2021; 7(8):000612	Inadequate population details
Coppens J, Xavier BB, Vlaeminck J, Larsen J, Lammens C, Van Puyvelde S, et al. Genomic analysis of methicillin-resistant *Staphylococcus aureus* clonal complex 239 isolated from Danish patients with and without an international travel history. Front Microbiol 2022; 13:1016829	Inadequate population details
Cox GW, Avery BP, Parmley EJ, Irwin RJ, Reid-Smith RJ, Deckert AE, et al. A One Health Genomic Investigation of Gentamicin Resistance in *Escherichia coli* from Human and Chicken Sources in Canada, 2014 to 2017. Antimicrob Agents Chemother 2022; 66(10):e0067722	Inadequate population details
Cui F, Liu L, Miao X, Qian H, Lu S, Tian J, et al. Epidemiological and comparative genomic analyses of Multidrug-Resistant *Acinetobacter baumannii* collected between 2020 and 2022 in Liaocheng City, Shandong Province, China. Journal of Biosafety and Biosecurity 2023; 5(2):60-66	Inadequate population details
Cuningham W, Perera S, Coulter S, Nimmo GR, Yarwood T, Tong SYC, et al. Antibiotic resistance in uropathogens across northern Australia 2007-20 and impact on treatment guidelines. JAC Antimicrobial Resistance 2021; 3(3):dlab127	WGS not included
da Silva KE, Thi Nguyen TN, Boinett CJ, Baker S, Simionatto S. Molecular and epidemiological surveillance of polymyxin-resistant *Klebsiella pneumoniae* strains isolated from Brazil with multiple mgrB gene mutations. Int J Med Microbiol 2020; 310(7):151448	Two tertiary care hospitals (187 and 352 beds) - not a regional surveillance study

Full reference	Reason for exclusion
Daley DA, Coombs GW, Yee NWT, Shoby P, Mowlaboccus S. The Australian Group on Antimicrobial Resistance (Agar) 2020 Australian Enterococcal Surveillance Outcome Programme (Aesop). Journal of Global Antimicrobial Resistance 2022; 31(Supplement 1):S8	Abstract
David S, Mentasti M, Sands K, Portal E, Graham L, Watkins J, et al. Genomic surveillance of carbapenem-resistant *Klebsiella* in Wales reveals persistent spread of *K. pneumoniae* ST307 and adaptive evolution of pOXA-48-like plasmids. bioRxiv 2022;	Inadequate population details
Decano AG, Pettigrew K, Sabiiti W, Sloan DJ, Neema S, Bazira J, et al. Pan-Resistome Characterization of Uropathogenic *Escherichia coli* and *Klebsiella pneumoniae* Strains Circulating in Uganda and Kenya, Isolated from 2017-2018. Antibiotics 2021; 10:1547	Inadequate population details
Del Barrio-Tofino E, Sanchez-Diener I, Zamorano L, Cortes-Lara S, Lopez-Causape C, Cabot G, et al. Association between Pseudomonas aeruginosa O-antigen serotypes, resistance profiles and high-risk clones: results from a Spanish nationwide survey. J Antimicrob Chemother 2019; 74(11):3217-3220	Inadequate population details
Deng X, Memari N, Teatero S, Athey T, Isabel M, Mazzulli T, et al. Whole-genome Sequencing for Surveillance of Invasive Pneumococcal Diseases in Ontario, Canada: Rapid Prediction of Genotype, Antibiotic Resistance and Characterization of Emerging Serotype 22F. Front Microbiol 2016; 7:2099	Non-protocol pathogen
Dereeper A, Gruel G, Pot M, Couvin D, Barbier E, Bastian S, et al. Limited Transmission of *Klebsiella pneumoniae* among Humans, Animals, and the Environment in a Caribbean Island, Guadeloupe (French West Indies). Microbiol 2022; 10(5):e0124222	Inadequate population details
Deshpande LM, Ashcraft DS, Kahn HP, Pankey G, Jones RN, Farrell DJ, et al. Detection of a New cfr-Like Gene, cfr(B), in Enterococcus faecium Isolates Recovered from Human Specimens in the United States as Part of the SENTRY Antimicrobial Surveillance Program. Antimicrob Agents Chemother 2015; 59(10):6256-61	Based on two patients, so not a surveillance study
Deshpande LM, Castanheira M, Flamm RK, Mendes RE. Evolving oxazolidinone resistance mechanisms in a worldwide collection of enterococcal clinical isolates: results from the SENTRY Antimicrobial Surveillance Program. J Antimicrob Chemother 2018; 73(9):2314-2322	Inadequate population details
Deshpande LM, Burian K, Doczi Csanyi I, Castanheira M. Dual VIM-producing Proteus mirabilis, Including a Novel VIM-75, Among Elderly Patients in a Medical Center from Hungary: Report from the 2020 SENTRY Antimicrobial Surveillance Program. Open Forum Infect Dis 2022; 9(Supplement 2):S811-S812	Abstract
Di Gregorio S, Vielma J, Haim MS, Rago L, Campos J, Kekre M, et al. Genomic epidemiology of S. aureus isolated from bloodstream infections in South America during 2019 supports regional surveillance. medRxiv 2022;	Inadequate population details
Diallo OO, Baron SA, Abat C, Colson P, Chaudet H, Rolain JM. Antibiotic resistance surveillance systems: A review. Journal of Global Antimicrobial Resistance 2020; 23:430-438	Review document
Dias JB, Soncini JGM, Cerdeira L, Lincopan N, Vespero EC. MDR *Escherichia coli* carrying CTX-M-24 (IncF[F-:A1:B32]) and KPC-2 (IncX3/IncU) plasmids isolated from community-acquired urinary trainfection in Brazil. Braz J Infect Dis 2022; 26(6):102706	Inadequate population details

Full reference	Reason for exclusion
Diorio-Toth L, Irum S, Potter RF, Wallace MA, Arslan M, Munir T, et al. Genomic Surveillance of Clinical Pseudomonas aeruginosa Isolates Reveals an Additive Effect of Carbapenemase Production on Carbapenem Resistance. Microbiol 2022; 10(3):e0076622	Inadequate population details
Doyle RM, O'Sullivan D M, Aller SD, Bruchmann S, Clark T, Pelegrin AC, et al. Discordant bioinformatic predictions of antimicrobial resistance from whole-genome sequencing data of bacterial isolates: An inter-laboratory study. Microbial Genomics 2020; 6(2)	Insufficient evidence that the study describes a surveillance system - isolates from just two hospitals so not operating at a regional or national level
Du P, Liu C, Fan S, Baker S, Guo J. The Role of Plasmid and Resistance Gene Acquisition in the Emergence of ST23 Multi-Drug Resistant, Hypervirulent *Klebsiella pneumoniae*. Microbiol 2022; 10(2):e0192921	Inadequate population details for the whole sample (except two patients)
Duffy N, Karlsson M, Reses HE, Campbell D, Daniels J, Stanton RA, et al. Epidemiology of extended-spectrum beta-lactamase-producing *Enterobacterales* in five US sites participating in the Emerging Infections Program, 2017. Infect Control Hosp Epidemiol 2022; 43(11):1586-1594	Pathogen was Enterobacterales, but not carbapenem resistant
Dwiyanto J, Hor JW, Reidpath D, Su TT, Lee SWH, Ayub Q, et al. Pan-genome and resistome analysis of extended-spectrum s-lactamase-producing *Escherichia coli*: A multi-setting epidemiological surveillance study from Malaysia. PLoS One 2022; 17(3):e0265142	Surveillance on too small a scale (one hospital and one small town)
Egli A, Blanc DS, Greub G, Keller PM, Lazarevic V, Lebrand A, et al. Improving the quality and workflow of bacterial genome sequencing and analysis: paving the way for a Switzerland-wide molecular epidemiological surveillance platform. Swiss Med Wkly 2018; 148:w14693	Commentary on ideal system but not reporting on an actual system that is operating
Eigenbrod T, Reuter S, Gross A, Kocer K, Gunther F, Zimmermann S, et al. Molecular characterization of carbapenem-resistant *Acinetobacter baumannii* using WGS revealed missed transmission events in Germany from 2012-15. J Antimicrob Chemother 2019; 74(12):3473-3480	Single hospital only
Elbadawi LI, Borlaug G, Gundlach KM, Monson T, Warshauer D, Walters MS, et al. Carbapenem-Resistant Enterobacteriaceae Transmission in Health Care Facilities - Wisconsin, February-May 2015. MMWR Morb Mortal Wkly Rep 2016; 65(34):906-9	Population details only for four patients
Ellaby N, Doumith M, Hopkins KL, Woodford N, Ellington MJ. Emergence of diversity in carbapenemase-producing *Escherichia coli* ST131, England, January 2014 to June 2016. Euro Surveill 2019; 24(37):1800627	Inadequate population details
Enstrom J, Froding I, Giske CG, Ininbergs K, Bai X, Sandh G, et al. USA300 methicillin-resistant *Staphylococcus aureus* in Stockholm, Sweden, from 2008 to 2016. PLoS One 2018; 13(11):e0205761	Inadequate population details
Estabrook M, Kazmierczak K, Arhin F, Sahm DF. In Vitro Activity of Aztreonam-Avibactam against *Klebsiella pneumoniae* Isolates Analyzed by Epidemic Lineage and Hypervirulence Factors Collected in China as Part of the ATLAS Global Surveillance Study in 2019. Open Forum Infect Dis 2021; 8(SUPPL 1):S721	Conference poster
Ferdous M, Zhou K, de Boer RF, Friedrich AW, Kooistra-Smid AM, Rossen JW. Comprehensive Characterization of *Escherichia coli* O104:H4 Isolated from Patients in the Netherlands. Front Microbiol 2015; 6:1348	Not a surveillance study

Full reference	Reason for exclusion
Ferreira FA, Helmersen K, Visnovska T, Jorgensen SB, Aamot HV. Rapid nanopore-based DNA sequencing protocol of antibiotic-resistant bacteria for use in surveillance and outbreak investigation. Microbial Genomics 2021; 7(4):000557	Inadequate population details
Findlay J, Nordmann P, Bouvier M, Kerbol A, Poirel L. Dissemination of ArmA- and OXA-23-co-producing *Acinetobacter baumannii* Global Clone 2 in Switzerland, 2020-2021. Eur J Clin Microbiol Infect Dis 2023;	Inadequate population details
Flores-Valdez M, Ares MA, Rosales-Reyes R, Torres J, Giron JA, Weimer BC, et al. Whole Genome Sequencing of Pediatric *Klebsiella pneumoniae* Strains Reveals Important Insights Into Their Virulence-Associated Traits. Front Microbiol 2021; 12:711577	Not a surveillance study
Fonseca EL, Caldart RV, Freitas FS, Morgado SM, Rocha LT, Dos Santos RC, et al. Emergence of extensively drug-resistant international clone IC-6 *Acinetobacter baumannii* carrying blaOXA-72 and blaCTX-M-115 in the Brazilian Amazon region. Journal of Global Antimicrobial Resistance 2020; 20:18-21	Not a surveillance study
Food Standards Agency. Enhanced molecular-based (MLST/whole genome) surveillance and source attribution of Campylobacter infections in the UK (FS101013) [Internet]. Oxford: Oxford University, 2021 Available from: https://doi.org/10.46756/sci.fsa.ksj135	Pathogen is campylobacter, which is not part of the inclusion criteria
Fuga B, Sellera FP, Cerdeira L, Esposito F, Cardoso B, Fontana H, et al. WHO Critical Priority *Escherichia coli* as One Health Challenge for a Post-Pandemic Scenario: Genomic Surveillance and Analysis of Current Trends in Brazil. Microbiol 2022; 10(2):e0125621	Inadequate population details
Fukuda A, Nakamura H, Umeda K, Yamamoto K, Hirai Y, Usui M, et al. Seven-year surveillance of the prevalence of antimicrobial-resistant *Escherichia coli* isolates, with a focus on ST131 clones, among healthy people in Osaka, Japan. Int J Antimicrob Agents 2021; 57(3):106298	Inadequate population details
FWD AMR-RefLabCap. Proposed protocol for whole genome sequencing-based analysis for detection and tracing of epidemic clones of antimicrobial resistant Salmonella and Campylobacter - to be used for national surveillance and integrated outbreak investigations by NRLs for public health, 2022 [accessed 8.9.23] Available from: https://www.fwdamr-reflabcap.eu/-/media/arkiv/projekt-sites/fwdamrreflabcap/resources/protocols-and-guidelines/fwd-amr-reflabcap-wgs-protocol-8-july-2022.pdf	Protocol for a proposed system rather than a report of an actual system in operation
Gagetti P, Lo SW, Hawkins PA, Gladstone RA, Regueira M, Faccone D, et al. Population genetic structure, serotype distribution and antibiotic resistance of streptococcus *pneumoniae* causing invasive disease in children in Argentina. Microbial Genomics 2021; 7(9):000636	Inadequate population details
Gali KV, St Jacques RM, Daniels CID, O'Rourke A, Turner L. Surveillance of carbapenem-resistant organisms using next-generation sequencing. Front Public Health 2023; 11:1184045	Inadequate population details
Gao Y, Li H, Chen H, Zhang J, Wang R, Wang Z, et al. Origin, Phylogeny, and Transmission of the Epidemic Clone ST208 of Carbapenem-Resistant *Acinetobacter baumannii* on a Global Scale. Microbiol 2022; 10(3):e0260421	Inadequate population details
Garcia-Castillo M, Garcia-Fernandez S, Gomez-Gil R, Pitart C, Oviano M, Gracia-Ahufinger I, et al. Activity of ceftazidime-avibactam against carbapenemase-producing Enterobacteriaceae from urine specimens obtained during	Inadequate population details

Full reference	Reason for exclusion
the infection-carbapenem resistance evaluation surveillance trial (iCREST) in Spain. Int J Antimicrob Agents 2018; 51(3):511-515	
Garcia-Gonzalez N, Beamud B, Fuster B, Giner S, Dominguez MV, Sanchez A, et al. Tracking the Emergence and Dissemination of a blaNDM-23 Gene in a Multidrug Resistance Plasmid of *Klebsiella pneumoniae*. Microbiol 2023; 11(2):e0258522	Poor reporting of data; in particular, unclear how many patients had WGS
Garretto A, Miller-Ensminger T, Ene A, Merchant Z, Shah A, Gerodias A, et al. Genomic Survey of *E. coli* From the Bladders of Women With and Without Lower Urinary Tract Symptoms. Front Microbiol 2020; 11:2094	Not a surveillance study
Garza-Gonzalez E, Franco-Cendejas R, Morfin-Otero R, Echaniz-Aviles G, Rojas-Larios F, Bocanegra-Ibarias P, et al. The Evolution of Antimicrobial Resistance in Mexico During the Last Decade: Results from the INVIFAR Group. Microbial Drug Resistance-Mechanisms Epidemiology & Disease 2020; 26(11):1372-1382	Inadequate population details
Geetha N, Bhaskaran M, Shamanna V, Marathe S, Ravikumar KL. Diverse Genetic Background of Multidrug Resistant Pseudomonas Aeruginosa Circulating in India. Int J Infect Dis 2023; 130(Supplement 2):S1-S2	conference abstract
Giani T, Antonelli A, Sennati S, Di Pilato V, Chiarelli A, Cannatelli A, et al. Results of the Italian infection-Carbapenem Resistance Evaluation Surveillance Trial (iCREST-IT): activity of ceftazidime/avibactam against *Enterobacterales* isolated from urine. J Antimicrob Chemother 2020; 75(4):979-983	No details of population characteristics
Gill CM, Akta E, Alfouzan W, Bourassa L, Brink A, Burnham CD, et al. The ERACE-PA Global Surveillance Program: Ceftolozane/tazobactam and Ceftazidime/avibactam in vitro Activity against a Global Collection of Carbapenem-resistant Pseudomonas aeruginosa. Eur J Clin Microbiol Infect Dis 2021; 40(12):2533-2541	Non-protocol pathogen
Gladstone RA, McNally A, Pontinen AK, Tonkin-Hill G, Lees JA, Skyten K, et al. Emergence and dissemination of antimicrobial resistance in *Escherichia coli* causing bloodstream infections in Norway in 2002-17: a nationwide, longitudinal, microbial population genomic study. Lancet Microbe 2021; 2(7):e331-e341	Duplicate of #2058
Gladstone RA, McNally A, Pontinen AK, Tonkin-Hill G, Lees JA, Skyten K, et al. Emergence and dissemination of antimicrobial resistance in *Escherichia coli* causing bloodstream infections in Norway in 2002-17: a nationwide, longitudinal, microbial population genomic study. Lancet Microbe 2021; 2(7):e331-e341	No details of population characteristics
Golden AR, Adam HJ, Baxter M, Martin I, Demczuk W, Mulvey MR, et al. Whole genome characterization of Streptococcus *pneumoniae* from respiratory and blood cultures collected from Canadian hospitals before and after PCV-13 implementation in Canada: Focus on serotypes 22F and 33F from CANWARD 2007-2018. Vaccine 2021; 39(39):5474-5483	Streptococcus *pneumoniae*
Golden AR, Karlowsky JA, Walkty A, Baxter MR, Denisuik AJ, McCracken M, et al. Comparison of phenotypic antimicrobial susceptibility testing results and WGS-derived genotypic resistance profiles for a cohort of ESBL-producing *Escherichia coli* collected from Canadian hospitals: CANWARD 2007-18. J Antimicrob Chemother 2021; 76(11):2825-2832	No details of population characteristics
Gona F, Comandatore F, Battaglia S, Piazza A, Trovato A, Lorenzin G, et al. Comparison of core-genome MLST, coreSNP and PFGE methods for *Klebsiella pneumoniae* cluster analysis. Microbial Genomics 2020; 6(4):000347	No details of population characteristics

Full reference	Reason for exclusion
Gordon LG, Elliott TM, Forde B, Mitchell B, Russo PL, Paterson DL, et al. Budget impact analysis of routinely using whole-genomic sequencing of six multidrug-resistant bacterial pathogens in Queensland, Australia. BMJ OPEN 2021; 11(2):e041968	No details of population characteristics
Greig DR, Do Nascimento V, Olonade I, Swift C, Nair S, Jenkins C. Surveillance of antimicrobial resistant Shiga toxin-producing *E. coli* O157:H7 in England, 2016-2020. J Antimicrob Chemother 2023; 78(9):2263-2273	No details of population characteristics
Hackel M, Wise MGG, Sahm DF. Activity of Cefepime in Combination with Taniborbactam (formerly VNRX-5133) Against Pseudomonas aeruginosa from a Global 2018-2020 Surveillance Collection. Open Forum Infect Dis 2021; 8(SUPPL 1):S124	Abstract
Hadjirin NF, van Tonder AJ, Blane B, Lees JA, Kumar N, Delappe N, et al. Dissemination of carbapenemase-producing *Enterobacterales* in Ireland from 2012 to 2017: a retrospective genomic surveillance study. Microbial Genomics 2023; 9(3):000924	No details of population characteristics
Heinz E, Brindle R, Morgan-McCalla A, Peters K, Thomson NR. Caribbean multi-centre study of *Klebsiella pneumoniae* : whole-genome sequencing, antimicrobial resistance and virulence factors. Microbial Genomics 2019; 5(5):000266	No details of population characteristics
Hendriksen RS, Bortolaia V, Tate H, Tyson GH, Aarestrup FM, McDermott PF. Using genomics to track global antimicrobial resistance. Front Public Health 2019; 7:242	Review document
Hernandez-Garcia M, Garcia-Fernandez S, Garcia-Castillo M, Bou G, Cercenado E, Delgado-Valverde M, et al. WGS characterization of MDR *Enterobacterales* with different ceftolozane/tazobactam susceptibility profiles during the SUPERIOR surveillance study in Spain. JAC Antimicrobial Resistance 2020; 2(4):dlaa084	No details of population characteristics
Higgs C, Kumar LS, Stevens K, Strachan J, Sherry NL, Horan K, et al. Population structure, serotype distribution and antibiotic resistance of Streptococcus *pneumoniae* causing invasive disease in Victoria, Australia. Microbial Genomics 2023; 9(7):001070	Non-protocol pathogen
Holden D, Sylvester M, Crandall J, Xu F, Schneider EC, Watson HB, et al. Identification and Whole Genome Sequencing Analysis of an Oxacillinase (OXA)-48-like-producing *Acinetobacter baumannii* Outbreak in California, January-May 2021. Open Forum Infect Dis 2021; 8(SUPPL 1):S109-S110	Conference abstract
Holmes A, Allison L, Ward M, Dallman TJ, Clark R, Fawkes A, et al. Utility of Whole-Genome Sequencing of *Escherichia coli* O157 for Outbreak Detection and Epidemiological Surveillance. J Clin Microbiol 2015; 53(11):3565-73	Pathogen was Enterobacterales, but not carbapenem resistant
Hornsey M, Wareham DW. Effects of In vivo Emergent Tigecycline Resistance on the Pathogenic Potential of *Acinetobacter baumannii*. Sci Rep 2018; 8(1):4234	No details of population characteristics
Horwich-Scholefield S, Lloyd T, Varghese V, Yette E, Huang S, Pandori M. Population-based surveillance of carbapenem-resistant enterobacteriaceae (CRE) in alameda County, 2017-2020. Open Forum Infect Dis 2020; 7(SUPPL 1):S721-S722	Abstract

Full reference	Reason for exclusion
Horwich-Scholefield S, Lloyd T, Varghese V, Yette E, Huang S, Pandori M. Imipenem-Relebactam Susceptibility and Genotypic Characteristics of Carbapenem-Resistant *Enterobacterales* Identified during Population-Based Surveillance. Antimicrob Agents Chemother 2021; 65(11):e0228820	Inadequate population details
Hosaka Y, Yahara K, Clark A, Kitagawa H, Hisatsune J, Sugai M, et al. Surveillance of multi-drug resistance phenotypes in *Staphylococcus aureus* in Japan and correlation with whole-genome sequence findings. J Hosp Infect 2022; 123:34-42	Inadequate population details
Hu X, Yang L, Dong N, Lin Y, Zhang L, Wang X, et al. Dissemination of blaNDM-5 in *Escherichia coli* through the IncX3 Plasmid from Different Regions in China. Microbial Drug Resistance-Mechanisms Epidemiology & Disease 2022; 28(4):453-460	Unable to access
Hu H, Shi Q, Zhang P, Quan J, Han X, Zhao D, et al. Prevalence and molecular characteristics of colistin-resistant isolates among clinically isolated carbapenem-resistant *Klebsiella pneumoniae* in China. Int J Antimicrob Agents 2023; 62(2):106873	Inadequate population details
Huang YC, Chen CJ, Lauderdale TLY. Detection, spread and phylogeny of meticillin-resistant *Staphylococcus aureus* sequence type 45 in Taiwan. Microbial Genomics 2021; 7(4):000555	Insufficient population details (only age)
Ingle DJ, Goncalves da Silva A, Valcanis M, Ballard SA, Seemann T, Jennison AV, et al. Emergence and divergence of major lineages of Shiga-toxin-producing *Escherichia coli* in Australia. Microbial Genomics 2019; 5(5):000268	Inadequate population details
Janssen AB, van Hout D, Bonten MJM, Willems RJL, van Schaik W. Microevolution of acquired colistin resistance in Enterobacteriaceae from ICU patients receiving selective decontamination of the digestive tract. J Antimicrob Chemother 2020; 75(11):3135-3143	Not a true surveillance study - one ICU used as source of isolates
Jesumirhewe C, Cabal-Rosel A, Allerberger F, Springer B, Ruppitsch W. Genetic characterization of *Escherichia coli* and *Klebsiella* spp. from humans and poultry in Nigeria. Access Microbiology 2023; 5(7):000509	Not a true surveillance study - one hospital and one college used as source of isolates
Jiang M, Li H, Liu X, Shen N, Zhou Y, Song W, et al. Genomic Analysis Revealed the International and Domestic Transmission of Carbapenem-Resistant *Klebsiella pneumoniae* in Chinese Pediatric Patients. Microbiol 2023:e0321322	Single hospital
Jozefikova A, Valcek A, Soltys K, Novakova E, Bujdakova H. Persistence and multi-ward dissemination of vancomycin-resistant Enterococcus faecium ST17 clone in hospital settings in Slovakia 2017-2020. Int J Antimicrob Agents 2022; 59(4):106561	Inadequate population details
Juraschek K, Borowiak M, Tausch SH, Malorny B, Kasbohrer A, Otani S, et al. Outcome of Different Sequencing and Assembly Approaches on the Detection of Plasmids and Localization of Antimicrobial Resistance Genes in Commensal *Escherichia coli*. Microorganisms 2021; 9(3):598	Non-human isolates
Kaku N, Sasaki D, Ota K, Miyazaki T, Yanagihara *K.* Changing molecular epidemiology and characteristics of methicillin-resistant *Staphylococcus aureus* isolated from bloodstream infections: nationwide surveillance in Japan in 2019. medRxiv 2021;	Inadequate population details

Full reference	Reason for exclusion
Kaku N, Sasaki D, Ota K, Miyazaki T, Yanagihara *K.* Changing molecular epidemiology and characteristics of *MRSA* isolated from bloodstream infections: nationwide surveillance in Japan in 2019. J Antimicrob Chemother 2022; 77(8):2130-2141	Inadequate population details
Kandasamy R, Lo S, Gurung M, Carter MJ, Gladstone R, Lees J, et al. Effect of childhood vaccination and antibiotic use on pneumococcal populations and genome-wide associations with disease among children in Nepal: an observational study. Lancet Microbe 2022; 3(7):e503-e511	Not a surveillance study - evaluating effects of vaccines and antibiotics
Kaprou GD, Bergspica I, Alexa EA, Alvarez-Ordonez A, Prieto M. Rapid Methods for Antimicrobial Resistance Diagnostics. Antibiotics 2021; 10:209	Review document
Karlowsky JA, Lob SH, Kazmierczak KM, Badal RE, Young K, Motyl MR, et al. In Vitro Activity of Imipenem against Carbapenemase-Positive Enterobacteriaceae Isolates Collected by the SMART Global Surveillance Program from 2008 to 2014. J Clin Microbiol 2017; 55(6):1638-1649	Inadequate population details
Karlowsky JA, Hackel MA, Wise MG, Six DA, Uehara T, Daigle DM, et al. In Vitro Activity of Cefepime-Taniborbactam and Comparators against Clinical Isolates of Gram-Negative Bacilli from 2018 to 2020: Results from the Global Evaluation of Antimicrobial Resistance via Surveillance (GEARS) Program. Antimicrob Agents Chemother 2023; 67(1):e0128122	Inadequate population details
Karlsson M, Lutgring JD, Ansari U, Lawsin A, Albrecht V, McAllister G, et al. Molecular Characterization of Carbapenem-Resistant *Enterobacterales* Collected in the United States. Microbial Drug Resistance-Mechanisms Epidemiology & Disease 2022; 28(4):389-397	Inadequate population details
Kawamoto Y, Kaku N, Akamatsu N, Sakamoto K, Kosai K, Morinaga Y, et al. The surveillance of colistin resistance and mobilized colistin resistance genes in multidrug-resistant Enterobacteriaceae isolated in Japan. Int J Antimicrob Agents 2022; 59(1):106480	Inadequate population details
Kayani H, Rasheed MA, Alonazi WB, Jamil F, Hussain A, Yan C, et al. Identification and genome-wide analysis provide insights into the genetic diversity and biotechnological potentials of novel cold-adapted *Acinetobacter* strain. Extremophiles 2023; 27(2):14	Not available at British Library (only held to 2018)
Kerdsin A, Deekae S, Chayangsu S, Hatrongjit R, Chopjitt P, Takeuchi D, et al. Genomic characterization of an emerging blaKPC-2 carrying Enterobacteriaceae clinical isolates in Thailand. Sci Rep 2019; 9(1):18521	Not a true surveillance study
Kim D, Ahn JY, Lee CH, Jang SJ, Lee H, Yong D, et al. Increasing Resistance to Extended-Spectrum Cephalosporins, Fluoroquinolone, and Carbapenem in Gram-Negative Bacilli and the Emergence of Carbapenem Non-Susceptibility in *Klebsiella pneumoniae*: Analysis of Korean Antimicrobial Resistance Monitoring System (KARMS) Data From 2013 to 2015. Annals of Laboratory Medicine 2017; 37(3):231-239	Inadequate population details
Kim YJ, Kim S, Kim J, Bae S. Tracking short-term changes in the genetic diversity and antimicrobial resistance of OXA-232-producing *Klebsiella pneumoniae* ST14 in clinical settings. Clin Microbiol Infect 2020; 26(1):78-86	Inadequate population details
Kim D, Yoon EJ, Hong JS, Choi MH, Kim HS, Kim YR, et al. Major Bloodstream Infection-Causing Bacterial Pathogens and Their Antimicrobial Resistance in South Korea, 2017-2019: Phase I Report From Kor-GLASS. Front Microbiol 2021; 12:799084	Inadequate population details

**Full reference**	**Reason for exclusion**
Kiyaga S, Kyany'a C, Muraya AW, Smith HJ, Mills EG, Kibet C, et al. Genetic Diversity, Distribution, and Genomic Characterization of Antibiotic Resistance and Virulence of Clinical Pseudomonas aeruginosa Strains in Kenya. Front Microbiol 2022; 13:835403	Inadequate population details
Kjaer Hansen S, Andersen L, Detlefsen M, Holm A, Roer L, Antoniadis P, et al. Using core genome multilocus sequence typing (cgMLST) for vancomycin-resistant Enterococcus faecium isolates to guide infection control interventions and end an outbrea*K.* Journal of Global Antimicrobial Resistance 2021; 24:418-423	Not a true surveillance study
Kluytmans-van den Bergh MF, Rossen JW, Bruijning-Verhagen PC, Bonten MJ, Friedrich AW, Vandenbroucke-Grauls CM, et al. Whole-Genome Multilocus Sequence Typing of Extended-Spectrum-Beta-Lactamase-Producing Enterobacteriaceae. J Clin Microbiol 2016; 54(12):2919-2927	Inadequate population details
Kohira N, Hackel MA, Ishioka Y, Kuroiwa M, Sahm DF, Sato T, et al. Reduced susceptibility mechanism to cefiderocol, a siderophore cephalosporin, among clinical isolates from a global surveillance programme (SIDERO-WT-2014). Journal of Global Antimicrobial Resistance 2020; 22:738-741	Inadequate population details
Koser CU, Ellington MJ, Peacock SJ. Whole-genome sequencing to control antimicrobial resistance. Trends Genet 2014; 30(9):401-7	Review document
Kourna Hama M, Khan D, Laouali B, Okoi C, Yam A, Haladou M, et al. Pediatric Bacterial Meningitis Surveillance in Niger: Increased Importance of Neisseria meningitidis Serogroup C, and a Decrease in Streptococcus *pneumoniae* Following 13-Valent Pneumococcal Conjugate Vaccine Introduction. Clin Infect Dis 2019; 69(Suppl 2):S133-S139	Incorrect pathogens
Krapp F, Salvatierra G, Hinostroza N, Garcia C, Astocondor AL, Ochoa T, et al. Molecular diversity and resistance mechanisms of *Klebsiella pneumoniae* bloodstream infections in Peru. Open Forum Infect Dis 2022; 9(Supplement 2):S123	Abstract
Kyany'a C, Nyasinga J, Matano D, Oundo V, Wacira S, Sang W, et al. Phenotypic and genotypic characterization of clinical *Staphylococcus aureus* isolates from Kenya. BMC Microbiol 2019; 19(1):245	Inadequate population details
La Bella G, Lopizzo T, Lupo L, Angarano R, Curci A, Manti B, et al. In vitro activity of ceftazidime/avibactam against carbapenem-nonsusceptible *Klebsiella* penumoniae isolates collected during the first wave of the SARS-CoV-2 pandemic: a Southern Italy, multicenter, surveillance study. Journal of Global Antimicrobial Resistance 2022; 31:236-238	Inadequate population details
Lee YL, Lu MC, Shao PL, Lu PL, Chen YH, Cheng SH, et al. Nationwide surveillance of antimicrobial resistance among clinically important Gram-negative bacteria, with an emphasis on carbapenems and colistin: Results from the Surveillance of Multicenter Antimicrobial Resistance in Taiwan (SMART) in 2018. Int J Antimicrob Agents 2019; 54(3):318-328	Inadequate population details
Lee YL, Ko WC, Lee WS, Lu PL, Chen YH, Cheng SH, et al. In-vitro activity of cefiderocol, cefepime/zidebactam, cefepime/enmetazobactam, omadacycline, eravacycline and other comparative agents against carbapenem-nonsusceptible *Enterobacterales*: results from the Surveillance of Multicenter Antimicrobial Resistance in Taiwan (SMART) in 2017-2020. Int J Antimicrob Agents 2021; 58(3):106377	Inadequate population details

Full reference	Reason for exclusion
Lee W, Williams P, Avison M. The Genomic Population Study of Bloodstream Associated *Escherichia coli* in 2020 in Southwest, U*K.* Int J Infect Dis 2023; 130(Supplement 2):S2-S3	Conference abstract
Lewis JM, Mphasa M, Banda R, Beale MA, Mallewa J, Anscome C, et al. Genomic analysis of extended-spectrum beta-lactamase (ESBL) producing *Escherichia coli* colonising adults in Blantyre, Malawi reveals previously undescribed diversity. Microbial Genomics 2023; 9(6):001035	Not a true surveillance study
Li F, Ye K, Li X, Ye L, Guo L, Wang L, et al. Genetic characterization of Carbapenem-Resistant *Escherichia coli* from China, 2015-2017. BMC Microbiol 2021; 21(1):248	No details of population characteristics
Li T, Yang Y, Yan R, Lan P, Liu H, Fu Y, et al. Comparing core-genome MLST with PFGE and MLST for cluster analysis of carbapenem-resistant *Acinetobacter baumannii*. Journal of Global Antimicrobial Resistance 2022; 30:148-151	No details of population characteristics
Li Z, Li Z, Peng Y, Lu X, Kan B. Trans-Regional and Cross-Host Spread of mcr-Carrying Plasmids Revealed by Complete Plasmid Sequences - 44 Countries, 1998-2020. China CDC Weekly 2022; 4(12):242-248	No details of population characteristics
Li X, Jiang T, Wu C, Kong Y, Ma Y, Wu J, et al. Molecular epidemiology and genomic characterization of a plasmid-mediated mcr-10 and blaNDM-1 co-harboring multidrug-resistant Enterobacter asburiae. Comput Struct Biotechnol J 2023; 21:3885-3893	No details of population characteristics
Liao CH, Lee NY, Tang HJ, Lee SS, Lin CF, Lu PL, et al. Antimicrobial activities of ceftazidime-avibactam, ceftolozane-tazobactam, and other agents against *Escherichia coli*, *Klebsiella pneumoniae* , and Pseudomonas aeruginosa isolated from intensive care units in Taiwan: results from the Surveillance of Multicenter Antimicrobial Resistance in Taiwan in 2016. Infect 2019; 12:545-552	No details of the population characteristics
Liao F, Mo Z, Gu W, Xu W, Fu X, Zhang Y. A comparative genomic analysis between methicillin-resistant *Staphylococcus aureus* strains of hospital acquired and community infections in Yunnan province of China. BMC Infect Dis 2020; 20(1):137	Data gained from only two hospitals - so not regional
Lim A, Naidenov B, Bates H, Willyerd K, Snider T, Couger MB, et al. Genomic surveillance for 1 antimicrobial resistance in mannheimia haemolytica using 2 nanopore single molecule sequencing technology. medRxiv 2018; https://doi.org/10.1101/395087	Bovine isolates of a non-protocol pathogen
Linkevicius M, Bonnin RA, Alm E, Svartstrom O, Apfalter P, Hartl R, et al. Rapid cross-border emergence of NDM-5-producing *Escherichia coli* in the European Union/European Economic Area, 2012 to June 2022. Euro Surveill 2023; 28(19):2300209	Inadequate population details
Lipworth S, Vihta KD, Chau K, Barker L, George S, Kavanagh J, et al. Ten-year longitudinal molecular epidemiology study of *Escherichia coli* and *Klebsiella* species bloodstream infections in Oxfordshire, U*K.* Genome Med 2021; 13(1):144	No details of population characteristics
Lipworth S, Vihta KD, Chau KK, Kavanagh J, Davies T, George S, et al. Ten Years of Population-Level Genomic *Escherichia coli* and *Klebsiella pneumoniae* Serotype Surveillance Informs Vaccine Development for Invasive Infections. Clin Infect Dis 2021; 73(12):2276-2282	Inadequate population details

**Full reference**	**Reason for exclusion**
Liu C, Chen K, Wu Y, Huang L, Fang Y, Lu J, et al. Epidemiological and genetic characteristics of clinical carbapenem-resistant *Acinetobacter baumannii* strains collected countrywide from hospital intensive care units (ICUs) in China. Emerg 2022; 11(1):1730-1741	No details of population characteristics
Liu C, Du P, Yang P, Zheng J, Yi J, Lu M, et al. Emergence and Inter- and Intrahost Evolution of Pandrug-Resistant *Klebsiella pneumoniae* Coharboring tmexCD1-toprJ1, blaNDM-1, and blaKPC-2. Microbiol 2023:e0278622	Single hospital
Lo SW, Gladstone RA, van Tonder AJ, Lees JA, du Plessis M, Benisty R, et al. Pneumococcal lineages associated with serotype replacement and antibiotic resistance in childhood invasive pneumococcal disease in the post-PCV13 era: an international whole-genome sequencing study. Lancet Infect Dis 2019; 19(7):759-769	No details of population characteristics
Love WJ, Zawack KA, Booth JG, Grohn YT, Lanzas C. Markov Networks of Collateral Resistance: National Antimicrobial Resistance Monitoring System Surveillance Results from *Escherichia coli* Isolates, 2004-2012. PLoS Comput Biol 2016; 12(11):e1005160	No details of population characteristics
Lu B, Lin C, Liu H, Zhang X, Tian Y, Huang Y, et al. Molecular Characteristics of *Klebsiella pneumoniae* Isolates From Outpatients in Sentinel Hospitals, Beijing, China, 2010-2019. Front Cell Infect Microbiol 2020; 10:85	No details of population characteristics
Ludden C, Raven KE, Jamrozy D, Gouliouris T, Blane B, Coll F, et al. One Health Genomic Surveillance of *Escherichia coli* Demonstrates Distinct Lineages and Mobile Genetic Elements in Isolates from Humans versus Livestoc*K.* mBio 2019; 10(1):e02693-18	Inadequate population details
Mancini S, Marchesi M, Imkamp F, Wagner K, Keller PM, Quiblier C, et al. Population-based inference of aminoglycoside resistance mechanisms in *Escherichia coli*. EBioMedicine 2019; 46:184-192	No details of population characteristics
Marin J, Clermont O, Royer G, Mercier-Darty M, Decousser JW, Tenaillon O, et al. The Population Genomics of Increased Virulence and Antibiotic Resistance in Human Commensal *Escherichia coli* over 30 Years in France. Applied & Environmental Microbiology 2022; 88(15):e0066422	Not a surveillance study - the isolates were from a collection taken over 30 years, and therefore not part of a current surveillance system operation
Marmor AM, Harley D. Evaluation of Australia's Enhanced Invasive Pneumococcal Disease (IPD) Surveillance Program. Commun Dis Intell 2018; 42	Commentary
Martin MJ, Stribling W, Ong AC, Maybank R, Kwak YI, Rosado-Mendez JA, et al. A panel of diverse *Klebsiella pneumoniae* clinical isolates for research and development. Microbial Genomics 2023; 9(5):000967	No details of population characteristics
Marusinec R, Shemsu M, Lloyd T, Kober BM, Herrera JA, Gregory M, et al. Epidemiology of Carbapenem-Resistant Organisms in Alameda County, California (July 2019 - June 2021). Open Forum Infect Dis 2022; 9(Supplement 2):S812-S813	Abstract
Mathur P, Malpiedi P, Walia K, Srikantiah P, Gupta S, Lohiya A, et al. Health-care-associated bloodstream and urinary tract infections in a network of hospitals in India: a multicentre, hospital-based, prospective surveillance study. Lancet Glob Health 2022; 10(9):e1317-e1325	No details of population characteristics

**Full reference**	**Reason for exclusion**
Mattioni Marchetti V, Kraftova L, Finianos M, Sourenian T, Hrabak J, Bitar I. Polyclonal Spread of Fosfomycin Resistance among Carbapenemase-Producing Members of the *Enterobacterales* in the Czech Republic. Microbiol 2023; 11(3):e0009523	No details of population characteristics
McDermott PF, Davis JJ. Predicting antimicrobial susceptibility from the bacterial genome: A new paradigm for one health resistance monitoring. Journal of Veterinary Pharmacology & Therapeutics 2021; 44(2):223-237	Review document
Medugu N, Tickler IA, Duru C, Egah R, James AO, Odili V, et al. Phenotypic and molecular characterization of beta-lactam resistant Multidrug-resistant *Enterobacterales* isolated from patients attending six hospitals in Northern Nigeria. Sci Rep 2023; 13(1):10306	No details of population characteristics
Mendes RE, Doyle TB, Critchley IA, Cotroneo N, Streit JM, Castanheira M. Molecular Epidemiology of *Escherichia coli* Causing Urinary Tract Infections in United States and in vitro Activity of Tebipenem, Including Against Strain Lineage and Resistant subsets (2018-2020). Open Forum Infect Dis 2021; 8(SUPPL 1):S715-S716	Conference abstract
Mendes RE, Arends SJR, Kimbrough JH, Kantro V, Butler D, Scangarella-Oman NE, et al. Activity of Gepotidacin Tested Against Molecularly Characterized *Escherichia coli* Isolates Resistant to Commonly Used Oral Therapies for UTI in the US (2019-2020). Open Forum Infect Dis 2022; 9(Supplement 2):S350	Conference abstract
Mendes RE, Hubler C, Kantro V, Shortridge D, Sader HS, Streit JM, et al. Cefiderocol In vitro Activity Against Molecularly Characterized Pseudomonas aeruginosa and *Acinetobacter baumannii*-calcoaceticus complex Clinical Isolates Causing Infection in United States Hospitals (2020-2021). Open Forum Infect Dis 2022; 9(Supplement 2):S351	Conference abstract
Mihai M, Manole A, Dascalu CG, Moraru D, Duca E, Manole M, et al. Surveillance Seasonal Program. Antibiotic Resistance of Pathogenic Agents Isolated From Children With Acute Gastroenteritis, in a Region of North-East Romania. Rev Med Chir Soc Med Nat Iasi 2016; 120(3):680-7	Insufficient methodology to ascertain this was a true surveillance study
Mills EG, Martin MJ, Luo TL, Ong AC, Maybank R, Corey BW, et al. A one-year genomic investigation of *Escherichia coli* epidemiology and nosocomial spread at a large US healthcare networ*K.* Genome Med 2022; 14(1):147	No details of population characteristics
Mitchell PK, Wang L, Stanhope BJ, Cronk BD, Anderson R, Mohan S, et al. Multi-laboratory evaluation of the Illumina iSeq platform for whole genome sequencing of Salmonella, *Escherichia coli* and Listeria. Microbial Genomics 2022; 8(2):000717	No details of population characteristics
Morgado S, Fonseca E, Vicente AC. Genomics of *Klebsiella pneumoniae* Species Complex Reveals the Circulation of High-Risk Multidrug-Resistant Pandemic Clones in Human, Animal, and Environmental Sources. Microorganisms 2022; 10(11):2281	No details of population characteristics
Morsli M, Boudet A, Kerharo Q, Stephan R, Salipante F, Dunyach-Remy C, et al. Real-time metagenomics-based diagnosis of community-acquired meningitis: A prospective series, southern France. EBioMedicine 2022; 84:104247	Not surveillance - point of care diagnostic testing
Mostafa HH, Cameron A, Taffner SM, Wang J, Malek A, Dumyati G, et al. Genomic Surveillance of Ceftriaxone-Resistant *Escherichia coli* in Western New York Suggests the Extended-Spectrum beta-Lactamase bla CTX-M-27 Is Emerging on Distinct Plasmids in ST38. Front Microbiol 2020; 11:1747	Inadequate population details

**Full reference**	**Reason for exclusion**
Mukherjee S, Blankenship HM, Rodrigues JA, Mosci RE, Rudrik JT, Manning SD. Antibiotic Susceptibility Profiles and Frequency of Resistance Genes in Clinical Shiga Toxin-Producing *Escherichia coli* Isolates from Michigan over a 14-Year Period. Antimicrob Agents Chemother 2021; 65(11):e0118921	Not a surveillance study - the isolates were from a collection taken over 14 years, and therefore not part of a current surveillance system operation
Mushtaq S, Reynolds R, Gilmore MC, Esho O, Adkin R, Garcia-Romero I, et al. Inherent colistin resistance in genogroups of the Enterobacter cloacae complex: epidemiological, genetic and biochemical analysis from the BSAC Resistance Surveillance Programme. J Antimicrob Chemother 2020; 75(9):2452-2461	Inadequate population details
Mustapha MM, Srinivasa VR, Griffith MP, Cho ST, Evans DR, Waggle K, et al. Genomic Diversity of Hospital-Acquired Infections Revealed through Prospective Whole-Genome Sequencing-Based Surveillance. mSystems 2022; 7(3):e0138421	Single hospital
Nabadda S, Kakooza F, Kiggundu R, Walwema R, Bazira J, Mayito J, et al. Implementation of the World Health Organization Global Antimicrobial Resistance Surveillance System in Uganda, 2015-2020: Mixed-Methods Study Using National Surveillance Data. JMIR Public Health and Surveillance 2021; 7(10):e29954	WGS not included
Nagaraj G, Govindan V, Ganaie F, Venkatesha VT, Hawkins PA, Gladstone RA, et al. Streptococcus *pneumoniae* genomic datasets from an Indian population describing pre-vaccine evolutionary epidemiology using a whole genome sequencing approach. Microbial Genomics 2021; 7(9):000645	Streptococcus *pneumoniae*
Nakano S, Fujisawa T, Ito Y, Chang B, Matsumura Y, Yamamoto M, et al. Whole-Genome Sequencing Analysis of Multidrug-Resistant Serotype 15A Streptococcus *pneumoniae* in Japan and the Emergence of a Highly Resistant Serotype 15A-ST9084 Clone. Antimicrob Agents Chemother 2019; 63(5)	Inadequate population details
Nguyen Q, Nguyen TTN, Pham P, Chau V, Nguyen LPH, Nguyen TD, et al. Genomic insights into the circulation of pandemic fluoroquinolone-resistant extra-intestinal pathogenic *Escherichia coli* ST1193 in Vietnam. Microbial Genomics 2021; 7(17):000733	Not a true surveillance study - two hospitals
NIHR Global Health Research Unit on Genomic Surveillance of AMR. Whole-genome sequencing as part of national and international surveillance programmes for antimicrobial resistance: a roadmap. BMJ Global Health 2020; 5:e002244	Review document
Nurjadi D, Zizmann E, Chanthalangsy Q, Heeg K, Boutin S. Integrative Analysis of Whole Genome Sequencing and Phenotypic Resistance Toward Prediction of Trimethoprim-Sulfamethoxazole Resistance in *Staphylococcus aureus*. Front Microbiol 2020; 11:607842	Inadequate population details
Octavia S, Xu W, Ng OT, Marimuthu K, Venkatachalam I, Cheng B, et al. Identification of AbaR4 *Acinetobacter baumannii* resistance island in clinical isolates of blaOXA-23-positive Proteus mirabilis. J Antimicrob Chemother 2020; 75(3):521-525	Inadequate population details
Odewale G, Jibola-Shittu MY, Ojurongbe O, Olowe RA, Olowe OA. Genotypic Determination of Extended Spectrum beta-Lactamases and Carbapenemase Production in Clinical Isolates of *Klebsiella pneumoniae* in Southwest Nigeria. Infectious Disease Reports 2023; 15(3):339-353	WGS not included

**Full reference**	**Reason for exclusion**
Odih EE, Oaikhena AO, Underwood A, Hounmanou YMG, Oduyebo OO, Fadeyi A, et al. High genetic diversity and blaNDM-1 prevalence among *Acinetobacter baumannii* in Nigerian hospitals. bioRxiv 2023;	Inadequate population details
Odih EE, Oaikhena AO, Underwood A, Hounmanou YMG, Oduyebo OO, Fadeyi A, et al. High Genetic Diversity of Carbapenem-Resistant *Acinetobacter baumannii* Isolates Recovered in Nigerian Hospitals in 2016 to 2020. mSphere 2023; 8(3):e0009823	Inadequate population details
Okeke IN, Aboderin AO, Egwuenu A, Underwood A, Afolayan AO, Kekre M, et al. Establishing a national reference laboratory for antimicrobial resistance using a whole-genome sequencing framework: Nigeria's experience. Microbiology 2022; 168(8):001208	Commentary on WGS systems, but inadequate population details
Oniciuc EA, Likotrafiti E, Alvarez-Molina A, Prieto M, Santos JA, Alvarez-Ordonez A. The present and future of whole genome sequencing (WGS) and whole metagenome sequencing (WMS) for surveillance of antimicrobial resistant microorganisms and antimicrobial resistance genes across the food chain. Genes (Basel) 2018; 9:268	Review document
Pajand O, Rahimi H, Darabi N, Roudi S, Ghassemi K, Aarestrup FM, et al. Arrangements of Mobile Genetic Elements among Virotype E Subpopulation of *Escherichia coli* Sequence Type 131 Strains with High Antimicrobial Resistance and Virulence Gene Content. mSphere 2021; 6(4):e0055021	Single referral hospital
Pataki BA, Matamoros S, van der Putten BCL, Remondini D, Giampieri E, Aytan-Aktug D, et al. Understanding and predicting ciprofloxacin minimum inhibitory concentration in *Escherichia coli* with machine learning. Sci Rep 2020; 10(1):15026	Inadequate population details
Pecora ND, Li N, Allard M, Li C, Albano E, Delaney M, et al. Genomically Informed Surveillance for Carbapenem-Resistant Enterobacteriaceae in a Health Care System. mBio 2015; 6(4):e01030	Single hospital
Pecora N, Zhao X, Nudel K, Hoffmann M, Li N, Onderdonk AB, et al. Diverse Vectors and Mechanisms Spread New Delhi Metallo-beta-Lactamases among Carbapenem-Resistant Enterobacteriaceae in the Greater Boston Area. Antimicrob Agents Chemother 2019; 63(2):e02040	Inadequate population details
Pedersen T, Tellevik MG, Kommedal O, Lindemann PC, Moyo SJ, Janice J, et al. Horizontal Plasmid Transfer among *Klebsiella pneumoniae* Isolates Is the Key Factor for Dissemination of Extended-Spectrum beta-Lactamases among Children in Tanzania. mSphere 2020;	Very narrow geographical scope, with three hospitals in one city - not a surveillance study
Peirano G, Matsumura Y, Adams MD, Bradford P, Motyl M, Chen L, et al. Genomic Epidemiology of Global Carbapenemase-Producing Enterobacter spp., 2008-2014. Emerg Infect Dis 2018; 24(6):1010-1019	No details of the population characteristics
Penven M, Zouari A, Nogues S, Collet A, Lecourt M, Birer A, et al. Web-based prediction of antimicrobial resistance in enterococcal clinical isolates by whole-genome sequencing. Eur J Clin Microbiol Infect Dis 2023; 42(1):67-76	Not available at British Library (only held to 2018)
Perez F, Bonomo RA. Insights into the evolving impact of multidrug resistant organisms (MDROs) through the integration of molecular and epidemiologic approaches. Clin Transl Sci 2013; Conference Publication:(var.pagings). 6(2):109	Conference abstract
Peri AM, Bauer MJ, Bergh H, Butkiewicz D, Paterson DL, Harris PN. Performance of the BioFire Blood Culture Identification 2 panel for the diagnosis of bloodstream infections. Heliyon 2022; 8(7):e09983	No details of the population characteristics

**Full reference**	**Reason for exclusion**
Perovic O, Singh-Moodley A, Lowe M. In Vitro Activity of Ceftolozane-Tazobactam against *Escherichia coli*, *Klebsiella pneumoniae* and Pseudomonas aeruginosa Obtained from Blood Cultures from Sentinel Public Hospitals in South Africa. Antibiotics 2023; 13:453	Not a surveillance study
Public Health England. Media backgrounder: Whole Genome Sequencing (WGS) and antibiotic-resistant bacteria [Internet], n.d. [accessed 8.9.23] Available from: https://assets.publishing.service.gov.uk/government/uploads/system/uploads/attachment_data/file/340026/template_AMR_v2.pdf	Media background information only
Plate A, Kronenberg A, Risch M, Mueller Y, Di Gangi S, Rosemann T, et al. Active surveillance of antibiotic resistance patterns in urinary tract infections in primary care in Switzerland. Infection 2019; 47(6):1027-1035	WGS not included
Probst K, Nurjadi D, Heeg K, Frede AM, Dalpke AH, Boutin S. Molecular Detection of Carbapenemases in *Enterobacterales*: A Comparison of Real-Time Multiplex PCR and Whole-Genome Sequencing. Antibiotics 2021; 10:726	Insufficient details of the population characteristics
Quino W, Caro-Castro J, Hurtado V, Flores-Leon D, Gonzalez-Escalona N, Gavilan RG. Genomic Analysis and Antimicrobial Resistance of Campylobacter jejuni and Campylobacter coli in Peru. Front Microbiol 2021; 12:802404	Insufficient details of the population characteristics
Rahmani A, Meradi L, Piris R. Molecular characterization of the whole genome in clinical multidrug-resistant strains of *Klebsiella pneumoniae*. J Infect Dev Ctries 2023; 17(1):59-65	Not a surveillance study
Rao M, Rashid FA, Shukor S, Hashim R, Ahmad N. Detection of Antimicrobial Resistance Genes Associated with Carbapenem Resistance from the Whole-Genome Sequence of *Acinetobacter baumannii* Isolates from Malaysia. Can J Infect Dis Med Microbiol 2020; 2020:5021064	Insufficient details of the population characteristics
Reslan L, Finianos M, Bitar I, Moumneh MB, Araj GF, Zaghlout A, et al. The Emergence of Invasive Streptococcus *pneumoniae* Serotype 24F in Lebanon: Complete Genome Sequencing Reveals High Virulence and Antimicrobial Resistance Characteristics. Front Microbiol 2021; 12:637813	Pathogen is streptococcus *pneumoniae*
Revez J, Espinosa L, Albiger B, Leitmeyer KC, Struelens MJ. Survey on the Use of Whole-Genome Sequencing for Infectious Diseases Surveillance: Rapid Expansion of European National Capacities, 2015-2016. Front Public Health 2017; 5:347	Survey of use of WGS rather than paper describing operation of a single WGS system
Rojas JD, Canal EA, Bernal MM, Lizewski RA, Rios PA. Characterization of circulating clinical multi-drug resistant and methicillin resistant *Staphylococcus aureus* isolates in Peru. Open Forum Infect Dis 2022; 9(Supplement 2):S115	Conference abstract
Saavedra SY, Bernal JF, Montilla-Escudero E, Torres G, Rodriguez MK, Hidalgo AM, et al. [National surveillance of clinical isolates of Enterococcus faecalis resistant to linezolid carrying the optrA gene in Colombia, 2014-2019]. Rev Panam Salud Publica 2020; 44:e104	Although there was one vancomycin-resistant isolate in study, this was not submitted for WGS
Said D, Willrich N, Ayobami O, Noll I, Eckmanns T, Markwart R. The epidemiology of carbapenem resistance in *Acinetobacter baumannii* complex in Germany (2014-2018): an analysis of data from the national Antimicrobial Resistance Surveillance system. Antimicrob 2021; 10(1):45	WGS not included

**Full reference**	**Reason for exclusion**
Said KB, Alsolami A, Khalifa AM, Khalil NA, Moursi S, Osman A, et al. A Multi-Point Surveillance for Antimicrobial Resistance Profiles among Clinical Isolates of Gram-Negative Bacteria Recovered from Major Ha'il Hospitals, Saudi Arabia. Microorganisms 2021; 9(10):2024	WGS not included
Salam MA, Al-Amin MY, Pawar JS, Akhter N, Lucy IB. Conventional methods and future trends in antimicrobial susceptibility testing. Saudi J Biol Sci 2023; 30(3):103582	Review document
Shaik S, Singh A, Suresh A, Ahmed N. Genome Informatics and Machine Learning-Based Identification of Antimicrobial Resistance-Encoding Features and Virulence Attributes in *Escherichia coli* Genomes Representing Globally Prevalent Lineages, Including High-Risk Clonal Complexes. mBio 2021; 13(1):e0379621	Inadequate population details
Shamanna V, Underwood A, Argimon S, Prasanna A, Nagaraj G, Govindan V, et al. Whole Genome Sequencing Reveals Diverse Modes of Carbapenemase Gene Dissemination in Indian *Klebsiella pneumoniae* Clinical Isolates. Int J Infect Dis 2023; 130(Supplement 2):S71-S72	Conference abstract
Shankar C, Jacob JJ, Sugumar SG, Natarajan L, Rodrigues C, Mathur P, et al. Distinctive Mobile Genetic Elements Observed in the Clonal Expansion of Carbapenem-Resistant *Klebsiella pneumoniae* in India. Microbial Drug Resistance-Mechanisms Epidemiology & Disease 2021; 27(8):1096-1104	Unable to access
Shelenkov A, Mikhaylova Y, Voskanyan S, Egorova A, Akimkin V. Whole-Genome Sequencing Revealed the Fusion Plasmids Capable of Transmission and Acquisition of Both Antimicrobial Resistance and Hypervirulence Determinants in Multidrug-Resistant *Klebsiella pneumoniae* Isolates. Microorganisms 2023; 11(5):1314	Not a surveillance study
Shen S, Han R, Yin D, Jiang B, Ding L, Guo Y, et al. A Nationwide Genomic Study of Clinical *Klebsiella pneumoniae* Carrying blaOXA-232 and rmtF in China. Microbiol 2023; 11(3):e0386322	Insufficient population details (only age)
Shorttidge D, Deshpande LM, Doyle TB, Streit JM, Castanheira M. Activity of meropenem-vaborbactam and comparators against globally disseminated *Klebsiella pneumoniae* sequence type 258. Open Forum Infect Dis 2020; 7(SUPPL 1):S791	Abstract
Sid Ahmed MA, Saleh TM, Albirair M, Dousa KM, Arisgado LC, Al Maslamani MA, et al. National Surveillance of Gram-negative Bacteria in Qatar, the Study for Monitoring of Antimicrobial Resistance Trends (SMART): from 2017 to 2019. Open Forum Infect Dis 2022; 9(Supplement 2):S664	Abstract
Sid Ahmed MA, Petkar HM, Saleh TM, Albirair M, Arisgado LA, Eltayeb FK, et al. The epidemiology and microbiological characteristics of infections caused by Gram-negative bacteria in Qatar: national surveillance from the Study for Monitoring of Antimicrobial Resistance Trends (SMART): 2017 to 2019. JAC Antimicrobial Resistance 2023; 5(4):dlad086	WGS not included
Simar SR, Hanson BM, Arias CA. Techniques in bacterial strain typing: past, present, and future. Curr Opin Infect Dis 2021; 34(4):339-345	Commentary on WGS techniques
Sirijatuphat R, Sripanidkulchai K, Boonyasiri A, Rattanaumpawan P, Supapueng O, Kiratisin P, et al. Implementation of global antimicrobial resistance surveillance system (GLASS) in patients with bacteremia. PLoS One 2018; 13(1):e0190132	Single hospital

Full reference	Reason for exclusion
Slown S, Walas N, Amato HK, Lloyd T, Varghese V, Bender M, et al. Clonal Lineages and Virulence Factors of Carbapenem Resistant *E. coli* in Alameda County, California, 2017-2019. Antibiotics 2022; 11:1794	No details of population characteristics
Spadar A, Phelan J, Elias R, Modesto A, Caneiras C, Marques C, et al. Genomic epidemiological analysis of *Klebsiella pneumoniae* from Portuguese hospitals reveals insights into circulating antimicrobial resistance. Sci Rep 2022; 12(1):13791	No details of population characteristics
Spaziante M, Venditti C, Butera O, Messina F, Di Caro A, Tonziello G, et al. Importance of Surveillance of New Delhi Metallo-Beta-Lactamase *Klebsiella pneumoniae*: Molecular Characterization and Clonality of Strains Isolated in the Lazio Region, Italy. Infect 2021; 14:3659-3665	No details of population characteristics
Stanton RA, Campbell D, McAllister GA, Breaker E, Adamczyk M, Daniels JB, et al. Whole-Genome Sequencing Reveals Diversity of Carbapenem-Resistant Pseudomonas aeruginosa Collected through CDC's Emerging Infections Program, United States, 2016-2018. Antimicrob Agents Chemother 2022; 66(9):e0049622	No details of population characteristics
Stewart A, Sarovich D, Price E, Burnard D, Harris P, Choong K, et al. Molecular epidemiology and hospital spread of ceftriaxone non-susceptible *enterobacterales* in the sunshine Coast Region, Australia. Intern Med J 2020; 50(Supplement 1):6-7	Abstract
Stoesser N, Phan HTT, Seale AC, Aiken Z, Thomas S, Smith M, et al. Genomic Epidemiology of Complex, Multispecies, Plasmid-Borne blaKPC Carbapenemase in *Enterobacterales* in the United Kingdom from 2009 to 2014. Antimicrob Agents Chemother 2020; 64(5):e02244	No details of population characteristics
Strommenger B, Ziegler S, Layer F, Bender J, Rothgaenger J, Harmsen D, et al. Towards NGS as frontline tool for routine typing of S. Aureus at the National Reference Center for staphylococci and *enterococci*? Int J Med Microbiol 2015; Conference Publication:(var.pagings). 305(SUPPL. 1):151	Conference abstract
Suaya JA, Mendes RE, Sings HL, Arguedas A, Reinert RR, Jodar L, et al. Streptococcus *pneumoniae* serotype distribution and antimicrobial nonsusceptibility trends among adults with pneumonia in the United States, 2009-2017. J Infect 2020; 81(4):557-566	Streptococcus *pneumoniae*
Sundermann AJ, Babiker A, Marsh JW, Shutt KA, Mustapha MM, Pasculle AW, et al. Outbreak of Vancomycin-resistant Enterococcus faecium in Interventional Radiology: Detection Through Whole-genome Sequencing-based Surveillance. Clin Infect Dis 2020; 70(11):2336-2343	Outbreak report
Suzuki S. A View on 20 Years of Antimicrobial Resistance in Japan by Two National Surveillance Systems: The National Epidemiological Surveillance of Infectious Diseases and Japan Nosocomial Infections Surveillance. Antibiotics 2021; 10:1189	No details of population characteristics
Takawira FT, Pitout JD, Thilliez G, Mashe T, Gutierrez AV, Kingsley RA, et al. Molecular epidemiology of extended-spectrum beta-lactamase-producing extra-intestinal pathogenic *Escherichia coli* strains over a 2-year period (2017-2019) from Zimbabwe. Eur J Clin Microbiol Infect Dis 2021;	Pathogen was Enterobacterales but not carbapenem-resistant
Takeuchi D, Kerdsin A, Akeda Y, Sugawara Y, Sakamoto N, Matsumoto Y, et al. Nationwide surveillance in Thailand revealed genotype-dependent dissemination of carbapenem-resistant *Enterobacterales*. Microbial Genomics 2022; 8(4):000797	No details of population characteristics

**Full reference**	**Reason for exclusion**
Talaat M, Zayed B, Tolba S, Abdou E, Gomaa M, Itani D, et al. Increasing Antimicrobial Resistance in World Health Organization Eastern Mediterranean Region, 2017-2019. Emerg Infect Dis 2022; 28(4):717-724	No details of population characteristics
Taylor E, Jauneikaite E, Sriskandan S, Woodford N, Hopkins KL. Detection and characterisation of 16S rRNA methyltransferase-producing Pseudomonas aeruginosa from the UK and Republic of Ireland from 2003-2015. Int J Antimicrob Agents 2022; 59(3):106550	Inadequate population details
Tian L, Sun Z, Zhang Z. Antimicrobial resistance of pathogens causing nosocomial bloodstream infection in Hubei Province, China, from 2014 to 2016: a multicenter retrospective study. BMC Public Health 2018; 18(1):1121	Inadequate population details
Timbrook TT, Olin KE, Spaulding U, Galvin BW, Cox CB. Epidemiology of Antimicrobial Resistance Among Blood and Respiratory Specimens in the United States Using Genotypic Analysis From a Cloud-Based Population Surveillance Networ*K.* Open Forum Infect Dis 2022; 9(7):ofac296	Inadequate population details
Toleman MS, Reuter S, Jamrozy D, Wilson HJ, Blane B, Harrison EM, et al. Prospective genomic surveillance of meticillinresistant *Staphylococcus aureus* (*MRSA*) associated with bloodstream infection, England, 1 October 2012 to 30 September 2013. Euro Surveill 2019; 24(4):1800215	Duplicate of #3501
Toth A, Lesinszki V, Janvari L, Kristof K, Kardos G, Topf J, et al. CHANGES in POPULATION STRUCTURE of MULTIDRUGRESISTANT *ACINETOBACTER BAUMANNII* in HUNGARY between 2010-2017. Acta Microbiol Immunol Hung 2018; 65(SUPPL 1):103	Conference abstract
Tsai CC, Lin JC, Chen PC, Liu EY, Tsai YK, Yu CP, et al. A 20-Year Study of Capsular Polysaccharide Seroepidemiology, Susceptibility Profiles, and Virulence Determinants of *Klebsiella pneumoniae* from Bacteremia Patients in Taiwan. Microbiol 2023; 11(3):e0035923	Inadequate population details
Ullah N, Dar HA, Naz K, Andleeb S, Rahman A, Saeed MT, et al. Genomic investigation of methicillin-resistant *staphylococcus aureus* st113 strains isolated from tertiary care hospitals in Pakistan. Antibiotics 2021; 10(9):1121	Inadequate population details; no evidence of this being a true surveillance study
Urbanowicz P, Izdebski R, Biedrzycka M, Literacka E, Hryniewicz W, Gniadkowski M. Genomic Epidemiology of MBL-Producing Pseudomonas putida Group Isolates in Poland. Infectious Diseases & Therapy 2022; 11(4):1725-1740	Inadequate population details
Uwanibe JN, Olawoye IB, Happi CT, Folarin OA. Genomic Characterisation of Multidrug-Resistant Pathogenic Enteric Bacteria from healthy children in Osun State, Nigeria. BioRxiv 2023;	Inadequate population details; does not appear to be a true surveillance study.
Valcek A, Nesporova K, Whiteway C, De Pooter T, De Coster W, Strazisar M, et al. Genomic Analysis of a Strain Collection Containing Multidrug-, Extensively Drug-, Pandrug-, and Carbapenem-Resistant Modern Clinical Isolates of *Acinetobacter baumannii*. Antimicrob Agents Chemother 2022; 66(9):e0089222	Inadequate population details
Varghese J, Chochua S, Tran T, Walker H, Li Z, Snippes Vagnone PM, et al. Multistate population and whole genome sequence-based strain surveillance of invasive pneumococci recovered in the USA during 2017. Clin Microbiol Infect 2020; 26(4):512.e1-512.e10	Insufficient details of the population characteristics.

**Full reference**	**Reason for exclusion**
Velasco JMS, Valderama MTG, Margulieux KR, Diones PCS, Reyes AMB, Leonardia SG, et al. First report of the mcr-1 colistin resistance gene identified in two *Escherichia coli* isolates from clinical samples, Philippines, 2018. Journal of Global Antimicrobial Resistance 2020; 21:291-293	Not a surveillance study
Vuichard-Gysin D, Harbarth S, Troillet N, Widmer A, Masserey V, Gardiol C. Successful collaboration between swissnoso and the federal office of public health (FOPH) during a national vancomycin resistant enterococcus faecium (*VRE*) outbrea*K.* Antimicrobial Resistance and Infection Control 2019; 8(Supplement 1):no pagination	Conference abstract
Waddington C, Carey ME, Boinett CJ, Higginson E, Veeraraghavan B, Baker S. Exploiting genomics to mitigate the public health impact of antimicrobial resistance. Genome Med 2022; 14(1):15	Commentary
Walas N, Slown S, Amato HK, Lloyd T, Bender M, Varghese V, et al. The role of plasmids in carbapenem resistant *E. coli* in Alameda County, California. BMC Microbiol 2023; 23(1):147	Insufficient details of the population characteristics
Walkty A, Karlowsky J, Lagace-Wiens P, Golden A, Baxter M, Denisuik A, et al. Presence of the Narrow-Spectrum OXA-1 Beta-lactamase Enzyme Is Associated with Elevated Piperacillin-Tazobactam MIC Values among ESBLproducing *Escherichia coli* Clinical Isolates (CANWARD, 2007-2018). Open Forum Infect Dis 2021; 8(SUPPL 1):S697	Conference abstract
Walkty A, Karlowsky J, Golden A, Lagace-Wiens P, Baxter M, Denisuik A, et al. Presence of the Narrow-Spectrum OXA-1 Beta-Lactamase Enzyme is not Associated with Elevated Ceftolozane-Tazobactam MIC Values among ESBL-Producing *Escherichia coli* Clinical Isolates (CANWARD, 2011-2018). Open Forum Infect Dis 2022; 9(Supplement 2):S363	Conference abstract
Wang J, Zhou M, Guo Z, Huang G, Zingg W. Surveillance of antimicrobial resistance in southern China in 2017. Antimicrobial Resistance and Infection Control 2019; 8(Supplement 1):no pagination	Conference abstract
Wang Y, Zhu B, Liu M, Dong X, Ma J, Li X, et al. Characterization of IncHI1B Plasmids Encoding Efflux Pump TmexCD2-ToprJ2 in Carbapenem-Resistant *Klebsiella* variicola, *Klebsiella* quasi*pneumoniae*, and *Klebsiella* michiganensis Strains. Front Microbiol 2021; 12:759208	Inadequate population details
Wang W, Baker M, Hu Y, Xu J, Yang D, Maciel-Guerra A, et al. Whole-Genome Sequencing and Machine Learning Analysis of *Staphylococcus aureus* from Multiple Heterogeneous Sources in China Reveals Common Genetic Traits of Antimicrobial Resistance. mSystems 2021; 6(3):e0118520	Inadequate population details
Wang B, Xu Y, Zhao H, Wang X, Rao L, Guo Y, et al. Methicillin-resistant *Staphylococcus aureus* in China: a multicentre longitudinal study and whole-genome sequencing. Emerg 2022; 11(1):532-542	Inadequate population details
Wang S, Wang L, Jin J, Li G, Shao H, Song Y, et al. Genomic Epidemiology and Characterization of Carbapenem-Resistant *Klebsiella pneumoniae* in ICU Inpatients in Henan Province, China: a Multicenter Cross-Sectional Study. Microbiol 2023; 11(3):e0419722	Inadequate population details
Weinmaier T, Conzemius R, Bergman Y, Lewis S, Jacobs EB, Tamma PD, et al. Validation and Application of Long-Read Whole-Genome Sequencing for Antimicrobial Resistance Gene Detection and Antimicrobial Susceptibility Testing. Antimicrob Agents Chemother 2023; 67(1):e0107222	Inadequate population details

Full reference	Reason for exclusion
Wise MGG, Kazmierczak K, Stone G, Sahm DF. In Vitro Activity of Ceftazidime-Avibactam and Comparators against KPCProducing *Enterobacterales* and Pseudomonas aeruginosa Collected in China as Part of the ATLAS Global Surveillance Program in 2019. Open Forum Infect Dis 2021; 8(SUPPL 1):S722-S723	Conference abstract
Wise MG, Karlowsky JA, Hackel MA, Takemura M, Yamano Y, Echols R, et al. In Vitro Activity of Cefiderocol Against Meropenem-Nonsusceptible Gram-Negative Bacilli with Defined beta-Lactamase Carriage: SIDERO-WT Surveillance Studies, 2014-2019. Microbial Drug Resistance-Mechanisms Epidemiology & Disease 2023; 29(8):360-370	Inadequate population details
Wohlfarth E, Kresken M, Higgins PG, Stefanik D, Wille J, Hafner D, et al. The evolution of carbapenem resistance determinants and major epidemiological lineages among carbapenem-resistant *Acinetobacter baumannii* isolates in Germany, 2010-2019. Int J Antimicrob Agents 2022; 60(5-6):106689	Inadequate population details
World Health Organization. GLASS: Whole-genome sequencing for surveillance of antimicrobial resistance. Geneva: WHO, 2020 [accessed 8.9.23] Available from: https://www.who.int/publications/i/item/9789240011007	Review document
Wu X, Shi Q, Shen S, Huang C, Wu H. Clinical and Bacterial Characteristics of *Klebsiella pneumoniae* Affecting 30-Day Mortality in Patients With Bloodstream Infection. Front Cell Infect Microbiol 2021; 11:688989	Not a surveillance study
Wu Y, Chen S, Li J, Cai C, Wang H, Zhou M, et al. Surveillance of Multidrug-Resistant Bacterial Infections in Non-Adult Patients - Zhejiang Province, China, 2014-2019. China CDC Weekly 2021; 3(47):1005-1013	WGS not included
Wyres KL, Nguyen TNT, Lam MMC, Judd LM, van Vinh Chau N, Dance DAB, et al. Genomic surveillance for hypervirulence and multi-drug resistance in invasive *Klebsiella pneumoniae* from South and Southeast Asia. Genome Med 2020; 12(1):11	Inadequate population details
Wysocka M, Monteiro T, de Pina C, Goncalves D, de Pina S, Ludgero-Correia A, et al. Whole-genome analysis uncovers loss of blaZ associated with carriage isolates belonging to methicillin-resistant *Staphylococcus aureus* (*MRSA*) clone ST5-VI in Cape Verde. Journal of Global Antimicrobial Resistance 2021; 26:77-83	Inadequate population details
Xie J, Liang B, Xu X, Yang L, Li H, Li P, et al. Identification of mcr-1-positive multidrug-resistant *Escherichia coli* isolates from clinical samples in Shanghai, China. Journal of Global Antimicrobial Resistance 2022; 29:88-96	Pathogen was Enterobacterales but not carbapenem-resistant
Yamba Yamba L, Udden F, Fuursted K, Ahl J, Slotved HC, Riesbeck *K.* Extensive/Multidrug-Resistant Pneumococci Detected in Clinical Respiratory Tract Samples in Southern Sweden Are Closely Related to International Multidrug-Resistant Lineages. Front Cell Infect Microbiol 2022; 12:824449	Insufficient population details (only age)
Yang C, Li Y, Zuo L, Jiang M, Zhang X, Xie L, et al. Genomic Epidemiology and Antimicrobial Susceptibility Profile of Enterotoxigenic *Escherichia coli* From Outpatients With Diarrhea in Shenzhen, China, 2015-2020. Front Microbiol 2021; 12:732068	Pathogen was Enterobacterales but not carbapenem-resistant
Yee R, Breitwieser FP, Hao S, Opene BNA, Workman RE, Tamma PD, et al. Metagenomic next-generation sequencing of rectal swabs for the surveillance of antimicrobial-resistant organisms on the Illumina Miseq and Oxford MinION platforms. Eur J Clin Microbiol Infect Dis 2021; 40(1):95-102	Inadequate population details
Yi M, Zou J, Zhao J, Tang Y, Yuan Y, Yang B, et al. Emergence of optrA-Mediated Linezolid Resistance in Enterococcus faecium: A Molecular Investigation in a Tertiary Hospital of Southwest China from 2014-2018. Infect 2022; 15:13-20	Not a surveillance study - isolates from one hospital

**Full reference**	**Reason for exclusion**
Zanichelli V, Huttner A, Harbarth S, Kronenberg A, Huttner B, Swiss Centre For Antibiotic Resistance A. Antimicrobial resistance trends in *Escherichia coli*, *Klebsiella pneumoniae* and Proteus mirabilis urinary isolates from Switzerland: retrospective analysis of data from a national surveillance network over an 8-year period (2009-2016). Swiss Med Wkly 2019; 149:w20110	WGS not included
Zelendova M, Papagiannitsis CC, Sismova P, Medvecky M, Pomorska K, Palkovicova J, et al. Plasmid-mediated colistin resistance among human clinical *Enterobacterales* isolates: national surveillance in the Czech Republic. Front Microbiol 2023; 14:1147846	Inadequate population details
Zhang X, Wang L, Li D, Li P, Yuan L, Yang F, et al. An IncP-2 plasmid sublineage associated with dissemination of blaIMP-45 among carbapenem-resistant Pseudomonas aeruginosa. Emerg 2021; 10(1):442-449	No details of population characteristics and does not fulfil conditions for surveillance study (only one tertiary hospital)
Zhang Y, Yang X, Liu C, Huang L, Shu L, Sun Q, et al. Increased clonal dissemination of OXA-232-producing ST15 *Klebsiella pneumoniae* in Zhejiang, China from 2018 to 2021. Infectious Diseases of Poverty 2023; 12(1):25	Inadequate population details
Zhou H, Zhang K, Chen W, Chen J, Zheng J, Liu C, et al. Epidemiological characteristics of carbapenem-resistant Enterobacteriaceae collected from 17 hospitals in Nanjing district of China. Antimicrob 2020; 9(1):15	Inadequate population details
Zhao Y, Zhang J, Fu Y, Li C, Hu K, Su S, et al. Molecular characterization of metallo-beta-lactamase- producing carbapenem-resistant Enterobacter cloacae complex isolated in Heilongjiang Province of China. BMC Infect Dis 2020; 20(1):94	No details of population characteristics
Zhu Z, Huang H, Wang M, Lv J, Xu L, Shi C, et al. Emergence and genomics of OXA-232-producing *Klebsiella pneumoniae* in a hospital in Yancheng, China. Journal of Global Antimicrobial Resistance 2021; 26:194-198	Not a surveillance study
Zhu C, Li C, Lai CKC, Ng R, Chau KY, Wong KT, et al. Longitudinal Genomic Characterization of Carbapenemase-producing Enterobacteriaceae (CPE) Reveals Changing Pattern of CPE Isolated in Hong Kong Hospitals. Int J Antimicrob Agents 2021; 58(5):106430	No details of population characteristics
